# Molidustat targets a synthetic lethal vulnerability in APC-mutant colorectal cancer through GSTP1 and PHD2 co-inhibition

**DOI:** 10.7554/eLife.110991

**Published:** 2026-11-10

**Authors:** Chiara Asselborn, Agata N Makar, Jair G Marques Junior, Aslihan Bastem Akan, Athanasia Yiapanas, Carrie Jennings, Ana Perez-Lopez, Jimi Wills, Asier Unciti-Broceta, Kevin B Myant, Alex von Kriegsheim

**Affiliations:** 1 Cancer Research UK Edinburgh Centre, Institute of Genetics and Cancer, University of Edinburgh Edinburgh United Kingdom; 2 Technische Universität Berlin, Si-M/'Der Simulierte Mensch', a Science Framework of Technische Universität Berlin and Charité – Universitätsmedizin Berlin Berlin Germany; University of Texas Southwestern Medical Center United States; Quest University Canada Canada

**Keywords:** colon organoids, RKO cells, HT29 cells, Human, Mouse

## Abstract

Mutations in the adenomatous polyposis coli (APC) gene are a defining feature of colorectal cancer (CRC) and impose metabolic and stress adaptation requirements that may create exploitable vulnerabilities. Prolyl hydroxylase domain (PHD) inhibitors have been explored as therapeutic agents in CRC; however, their mechanisms of action and off-target effects remain elusive. Serendipitously, we found that Molidustat, a PHD2 inhibitor, induced cell death in human APC-mutant CRC cells. Ablation of PHD2 alone did not affect cell viability, suggesting an off-target mechanism. Using thermal proteome profiling and chemical proteomics, we identify glutathione S-transferase P1 (GSTP1) as a previously unrecognised off-target of Molidustat and demonstrate direct inhibition of its enzymatic activity. Genetic ablation of PHD2 alone did not phenocopy the cytotoxic effects of Molidustat, whereas combined loss of PHD2 and GSTP1 induced synergistic proteomic changes associated with cell-cycle suppression and apoptotic signalling. Integrated proteomic and metabolomic analyses further revealed energetic and metabolic perturbations specific to simultaneous GSTP1 and PHD2 loss. Consistent with these findings, APC-mutant mouse organoids displayed selective sensitivity to Molidustat that was not reproduced by hydroxylase inhibition alone, supporting a synthetic lethal interaction between GSTP1 and PHD2 in APC-mutant contexts. Together, these results identify a functional interaction between GSTP1 and PHD2 in a subset of CRC and suggest that off-target engagement of GSTP1 contributes to the anti-tumour activity of Molidustat.

## Introduction

Colorectal cancer (CRC) is the second leading cause of cancer-related death worldwide and a major contributor to global cancer morbidity (Bray et al., 2024). Despite advances in screening and targeted therapy, long-term survival remains poor, underscoring the need to identify molecular vulnerabilities that can be exploited therapeutically. Understanding the adaptive mechanisms that enable tumour cells to tolerate oncogenic stress is critical for developing precision approaches to CRC treatment.

Mutations in the adenomatous polyposis coli (APC) gene occur in the vast majority of CRCs and represent the initiating event in tumour development (Zhang and Shay, 2017; Powell et al., 1992). Loss or mutation of APC leads to constitutive activation of the Wnt/β-catenin signalling pathway, driving uncontrolled proliferation (Morin et al., 1997; Korinek et al., 1997; Neufeld et al., 2000). Moreover, in APC-mutant cells, β-catenin signalling contributes to adaptation programmes that support tumour growth under low oxygen availability (hypoxia) (Kaidi et al., 2007). During hypoxia, oxygen sensing and signal transduction are largely governed by prolyl hydroxylase domain proteins (PHD1/2/3), which hydroxylate target proteins in an oxygen-dependent manner (Epstein et al., 2001; Bruick and McKnight, 2001). Canonically, PHDs control the stability of hypoxia-inducible factor-1α (HIF1α) by promoting its proteasomal degradation under normoxic conditions, in which molecular oxygen availability permits efficient hydroxylation and binding to VHL protein (Berra et al., 2003; Appelhoff et al., 2004). However, these hydroxylases also modify non-HIF substrates, and substrate-trapping proteomics has shown that hydroxylase signalling extends to pathways relevant to Wnt regulation (Rodriguez et al., 2016). Altogether these observations suggest that modulation of PHD activity may represent an attractive approach for targeting APC-deficient CRC.

Pharmacological inhibitors of PHDs are widely used to disrupt the hydroxylase function. The most frequently used compound, dimethyloxalylglycine (DMOG), is a pan-PHD inhibitor simultaneously disrupting the function of all 2-oxoglutarate-dependent dioxygenases (Tian et al., 2011; Zhdanov et al., 2015). In contrast, newer clinical compounds such as Molidustat (BAY 85-3934) were developed with improved selectivity towards distinct PHD enzymes and optimised pharmacological properties (Hirota, 2021). However, selective PHD inhibition can exert broader biological effects: IOX5 has been shown to suppress tumour growth and induce apoptosis in acute myeloid leukaemia through HIF-regulated and off-target mechanisms (Lawson et al., 2024). Likewise, although Molidustat has been shown to reduce tumour survival in breast cancer models, the mechanism of action and off-targets of this compound remain unknown (Kachamakova-Trojanowska et al., 2020).

Glutathione S-transferase P1 (GSTP1) is a detoxification enzyme that conjugates glutathione (GSH) to electrophilic substrates and modulates stress-activated signalling pathways (Bogaards et al., 1997; Board and Menon, 2013). GSTP1 is frequently upregulated in CRC and promotes proliferation and invasion through STAT3 activation (Wang et al., 2022). Altered GSTP1 expression or methylation has also been associated with tumour progression and therapy resistance in other cancers (Arai et al., 2008; Cui et al., 2020). Given its role in maintaining cellular stability under stress, GSTP1 may counteract the cytotoxic consequences that arise when oxygen-sensing pathways are perturbed. Consequently, GSTP1 inhibitors are an active area of research, with several compounds at the pre-clinical and clinical stage (Raza et al., 2009; Pereira et al., 2025; Wang et al., 2023).

Driven by the connection between hydroxylase activity, APC-relevant signalling pathways, and by observations that selective PHD inhibition can affect tumour cell survival, we investigated how PHD inhibition influences adaptive stress responses in APC-mutant CRC. In this study, we characterise cellular targets of the clinical PHD inhibitor Molidustat and identify a synthetic lethal interaction between GSTP1 and PHD2 inhibition in APC-mutant CRC models. Using pharmacological inhibition and CRISPR-mediated gene disruption, we demonstrate that concurrent blockade of GSTP1 and PHD2 synergistically disrupts homeostatic control, inducing proteomic reprogramming, accumulative stress, and apoptotic cell death in APC-deficient cells. This dual vulnerability exposes a therapeutic window for selectively targeting APC-mutant CRC while sparing normal epithelia.

## Methods

### Cell culture and treatments

Human CRC cell lines HT29 and RKO were obtained from the American Type Culture Collection (ATCC) and maintained in Dulbecco’s Modified Eagle Medium (DMEM; Sigma-Aldrich, D6429) supplemented with 10% (vol/vol) foetal bovine serum (Gibco, 16000044), 1% (vol/vol) penicillin-streptomycin (Gibco, 15140122), and 2 mM L-glutamine (Sigma-Aldrich, G7513). Cells were cultured at 37°C in a humidified incubator with 5% CO_2_. The parental lines were purchased from the ATCC. All cell lines were tested for mycoplasma contamination every 3 months by the Institute of Genetics and Cancer technical services and were confirmed to be negative throughout.

For chemical inhibition of PHDs, cells were treated with Molidustat (BAY 85-3934; Cayman Chemical, 17744), IOX4 (Tocris Bioscience, 5084), DMOG (Cayman Chemical, 71210), or JNJ-42041935 (Sigma-Aldrich, SML1390). For iron chelation-based HIF stabilisation, deferoxamine mesylate (DFO; Sigma-Aldrich, D9533) was used. For GSTP1 inhibition, Ezatiostat (Cayman Chemical, CAY-16248) was used. All compounds were dissolved in DMSO (Sigma-Aldrich, D2650) to prepare 10 mM stocks and diluted in complete medium to the indicated concentrations. Cells were seeded 24 hr prior to treatment to achieve 60–70% confluence and incubated with compounds or vehicle control for the specified durations.

### 3D organoid culture

Murine wild-type (WT) and *Apc^fl/fl^* organoids were generated as previously described (Sato et al., 2009). *Apc^fl/fl^* intestinal organoids were derived from Villin-CreER^T2^;*Apc^fl/fl^* mice induced with tamoxifen (120 mg/kg on day 0 and 80 mg/kg on day 1) and sacrificed on day 5, and WT organoids from C57BL/6 mice. Briefly, intestinal crypts were isolated from freshly dissected small intestines, washed in cold phosphate-buffered saline (PBS), and incubated in 2 mM EDTA (Thermo Fisher Scientific, 15575020) for 30 min on ice. Crypts were pelleted (300×*g*, 5 min, 4°C), resuspended in growth-factor-reduced Matrigel (Corning, 356231) or BME (Basement Membrane Extract Matrix, Type 2, Trevigen #3533-010-02), and plated in 24-well plates. After polymerisation (15 min, 37°C), organoids were overlaid with advanced DMEM/F12 (Gibco, 12634010) supplemented with 10 mM HEPES (Invitrogen, 15630106), 1% (vol/vol) penicillin-streptomycin (Gibco, 15140122), and 2 mM L-glutamine (Sigma-Aldrich, G7513), N2 (Invitrogen, 17502048), and B27 (Invitrogen, 17504044). Medium was also supplemented with EGF (Peprotech, 315-09-500UG), 1% conditioned medium Noggin (produced in-house). WT organoid medium was further supplemented with 10% conditioned medium R-Spondin (produced in-house). AKP (*Apc^fl/fl^;Kras^LSL-G12D/+^;Trp53^fl/fl^*) organoids, derived from the colon of non-induced mice and recombined in vitro with adenoviral Cre, were kindly provided by Dr Ömer Yilmaz (Massachusetts Institute of Technology). KPN (Villin-CreER^T2^;*Kras^G12D/+^;Trp53^fl/fl^;Rosa26^N1icd/+^*) organoids, derived from liver metastases, were kindly provided by Prof. Owen Sansom (Cancer Research UK Scotland Institute, Glasgow).

### In vivo Molidustat treatment

All mouse models were bred and kept in the animal facilities of the University of Edinburgh according to UK Home Office regulations. They were kept in 12 hr cycles of light/dark and had access to water and standard food ad libitum. All animal experiments were approved by the University of Edinburgh Animal Welfare and Ethical Review Body (AWERB) and were carried out under UK Home Office Project Licence 7008885 and Personal Licence I97725097, in accordance with the Animals (Scientific Procedures) Act 1986 and the ARRIVE guidelines.

Molidustat-treated (n=10; 4 male, 6 female) and vehicle-treated (n=10; 5 male, 5 female) mice were aged 17–25 weeks at tamoxifen induction and on a mixed background. Animals were allocated to treatment groups at random, and investigators were not blinded to group allocation. No animals were excluded from the analysis. Gene deletion in *Lgr5*-EGFP-ires-CreER^T2^;*Apc^fl/fl^* mice (Pickering et al., 2021) was induced using intraperitoneal (IP) injection of tamoxifen on 2 consecutive days (120 mg/kg on day 0 and 80 mg/kg on day 1). After induction with tamoxifen, mice were treated daily with Molidustat (10 mg/kg, suspended in 2.5% DMSO in PBS; administered by IP injection in 200 µl) and compared to vehicle-only controls. Once the animals reached the clinical endpoint (moderate severity, defined by weight loss, rectal bleeding, hunching, and pale feet), they were sacrificed by cervical dislocation. 2 hr prior, the mice were injected with 200 µl BrdU (GE Healthcare, RPN201). For IHC, tissues were first washed with PBS and fixed in 4% paraformaldehyde for 24 hr at 4°C. Tissues were processed in a Tissue-TEK VIP Infiltration Processor (Sakura). The processed samples were embedded in paraffin wax and 5 μm sections cut on a microtome (Leica). Standard immunohistochemistry and histology techniques were used. A 1/500 dilution was used for the BrdU antibodies (mouse monoclonal, clone B44; BD Biosciences, 347580; RRID:AB_400326; and rabbit polyclonal; Bioss, bs-0489R; RRID:AB_10856208). Images were taken using the Nanozoomer Digital slide scanner (Hamamatsu) with the NDP.view2 software (Hamamatsu). QuPath (Bankhead et al., 2017) was used for image analysis and quantification. For the qRT-PCR experiment, which was used to assess the expression of *Epo* mRNA in the kidney, SYBR Select Master Mix (Applied Biosystems, 4472920) was used according to the protocol and the reaction was performed in a CFX Connect Real-time System (Bio-Rad) machine. *Epo* forward: 5’-ACTCTCCTTGCTACTGATTCCT-3’, *Epo* reverse: 5’-ATCGTGACATTTTCTGCCTCC-3’.

### CRISPR/Cas9-mediated knockout

Gene knockouts were generated either using the Edit-R pre-designed crRNA (Dharmacon) system or the Alt-R CRISPR-Cas9 system (Integrated DNA Technologies [IDT]). For the Dharmacon system, HT29 and RKO cells were transduced with Lentiviral Cas9 (Addgene: #52962) and selected with blasticidine for at least 3 days. Pre-designed PHD2 (*EGLN1*) crRNAs (CM-004276-01-0002, CM-004276-02-0002, CM-004276-03-0002; targeting sequences: TACAACCAGCATATGCTACA, GTGGCTGCCGAAGCCGAGCC, GATAAGATCACCTGGATCGA, respectively) or *GSTP1* targeting sequences (AA GGGAAATAGACCACGGTGTA, AATACCATCCTGCGTCACCT) were mixed with tracrRNA (Dharmacon, U-002005-05) for 5 min, incubated with DharmaFECT 2 for 20 min, before transfecting the cells. Knockout efficiency was assessed after 3 days. Edit-R Non-targeting crRNA Control #1 (U-007501-01-05) and Edit-R Lethal crRNA Control #1 (U-006000-01-05) were used as controls. For the Alt-R CRISPR-Cas9 system (IDT), crRNAs targeting human or mouse PHD2 (*EGLN1*) and *GSTP1* were designed using the IDT design tool. crRNAs were annealed with tracrRNA (IDT, 1072533) by heating at 95°C for 5 min and cooling to room temperature. Cas9 nuclease (IDT, 1081058) was incubated with the RNA duplex for 15 min to form ribonucleoprotein complexes. Cells were nucleofected using the Amaxa 4D-Nucleofector system (Lonza, V4XC-1032) with the SE Cell Line Kit according to the manufacturer’s protocol. Transfected cells were recovered for 48 hr in complete medium before selection. Knockout efficiency was validated by western blotting.

### Caspase-3/7 activity assay

Apoptotic cell death was quantified using the CellEvent Caspase-3/7 Green Detection Reagent (Invitrogen, C10423). Cells were seeded in 96-well plates and treated with inhibitors or vehicle control. Caspase-3/7 reagent (2 µM or 5 µM final concentration) was added directly to the medium, and plates were imaged using the IncuCyte Live-Cell Analysis System (Essen Biotech). Caspase activity was quantified using IncuCyte software. In organoids, CellEvent Caspase-3/7 Green Detection Reagent (Invitrogen, C10423) was added directly to the medium (2 µM final concentration), and brightfield and fluorescent images were captured manually. Images were analysed using ImageJ (Fiji) software.

### MTT cell viability assay

Organoids were seeded in 96-well plates and cultured for 2 days before treatment with fresh medium containing PHD inhibitors or vehicle control. After 48 hr, the medium was removed and replaced with fresh medium containing a final concentration of 5 μg/ml MTT reagent (Sigma, M2128). After 2 hr incubation at 37°C, the MTT medium was removed and 2% sodium dodecyl sulphate (SDS) (in H_2_O) was added to solubilise the Matrigel. After 2 hr, DMSO was added to the wells to solubilise the reduced MTT. After 1 hr, the optical density was measured on a microplate reader (Wallac, 1420 Victor2 microplate reader) at 570 nm and 690 nm (background).

### Clonogenicity assay

Organoids were seeded in 24-well plates and cultured for 2 days before treatment with Molidustat or vehicle control. After 24 hr, organoids were collected, washed, and centrifuged at 300×*g* (3 min), followed by dissociation to single cells using TrypLE (Gibco, 12604013) for 20 min (37°C) and passed through a 40 µm cell strainer. 10,000 single cells were resuspended per 10 μl droplet of fresh BME (Trevigen #3533-010-02). The BME droplets were covered in standard organoid culture medium, supplemented with Y27632 dihydrochloride (ROCK inhibitor, Tocris, 1254). After 3 days, brightfield images of the resulting clones were captured and counted using ImageJ (Fiji) software.

### Lgr5 RT-qPCR

Organoids were cultured as described above for 2 days followed by treatment with Molidustat or vehicle control for 48 hr. Organoid RNA was extracted using the RNeasy Mini Kit (QIAGEN, 74106), and DNase treatment (Invitrogen, AM1906) was performed. cDNA was synthesised using 500 ng to 1 µg RNA and the qScript cDNA SuperMix (Quantabio, 95048-100). cDNA was diluted (1/10) prior to real-time quantitative polymerase chain reaction (RT-qPCR) on the CFX Connect Real-Time System (Bio-Rad). The reaction was prepared using SYBR Select Master Mix (Applied Biosystems, 4472920) and the following *Lgr5* oligonucleotides: 5’ GAGTCAACCCAAGCCTTAGTATCC 3’ and 5’ CATGGGACAAATGCAACTGAAG 3’.

### Protein extraction and western blotting

Cells were lysed in RIPA buffer (Thermo Fisher Scientific, 89901) supplemented with protease and phosphatase inhibitors (Roche, 11836170001 and 4906837001). Lysates were cleared by centrifugation (14,000×*g*, 15 min, 4°C) and quantified using the BCA Protein Assay Kit (Pierce, 23225). Equal protein amounts were mixed with 4× NuPage LDS buffer (Thermo Fisher Scientific, NP0007), denatured, and separated by 10% SDS-PAGE. Proteins were transferred to PVDF membranes (Millipore, IPVH00010), blocked in 5% non-fat milk/TBS-T, and incubated overnight at 4°C with primary antibodies diluted in 5% milk/TBS-T: PHD2 (clone D31E11; Cell Signaling Technology, 4835; RRID:AB_10561316; 1:1000), HIF1α (clone H1alpha67; Novus Biologicals, NB100-105; RRID:AB_10001154; 1:500), GSTP1 (clone 3F2C2; Santa Cruz Biotechnology, sc-66000; RRID:AB_2279559; 1:1000), β-tubulin (rabbit polyclonal; Cell Signaling Technology, 2146; RRID:AB_2210545; 1:1000), and β-actin (clone JJ09-29; Invitrogen, MA5-32479; RRID:AB_2809756; 1:2500). HRP-linked anti-rabbit IgG (7074; RRID:AB_2099233) or anti-mouse IgG (7076; RRID:AB_330924) secondary antibodies (1:10,000; Cell Signaling Technology) and either Clarity Western ECL substrate (Bio-Rad, 1705061) or SuperSignal West Femto (Thermo Scientific, 34094) were used for detection on a Bio-Rad ChemiDoc XRS+Imaging System.

### GSTP1 recombinant protein expression and enzymatic assays

The plasmid used for recombinant expression of human GSTP1 was kindly provided by Dr Anna-Maria Caccuri (University of Rome). Recombinant GSTP1 was expressed in *Escherichia coli* BL21 (DE3) cells induced with 0.5 mM IPTG (Sigma-Aldrich, I6758) for 4 hr at 30°C. Cells were lysed by sonication in PBS containing 1 mM PMSF (Sigma-Aldrich, P7626), and the fusion protein was purified using Glutathione Sepharose 4B resin (Cytiva, 17-0756-05). GSTP1 was eluted with 10 mM reduced GSH (Sigma-Aldrich, G4251). Enzymatic activity was measured spectrophotometrically at 340 nm using GSH and 1-chloro-2,4-dinitrobenzene (CDNB; Sigma-Aldrich, 138630) in 100 mM potassium phosphate buffer (pH 6.5). For inhibition assays, purified GSTP1 (0.5 µg) was pre-incubated with ethacrynic acid (EA; Sigma-Aldrich, E8750; 1–100 µM, 30 min, room temperature) or Molidustat (BAY 85-3934; Cayman Chemical, 17744) before substrate addition. Enzymatic activity was calculated as the change in absorbance per minute relative to control reactions.

### In silico docking

The conformational energy of the compounds was minimised using Avogadro v.1.2.0. The compounds were docked using a modified protein structure from the Protein Data Bank (https://www.rcsb.org/structure/5X79) using GOLD software in Hermes through Cambridge Crystallography Database (CCDC) v.2025.1. The highest docking scores were visualised in PyMol v.3.1.5.1, and the figures were generated using PyMol v.3.1.5.1.

### ROS and antioxidant treatment

Reactive oxygen species (ROS) were detected using the Peroxy Orange 1 (PO1) fluorescent probe (Tocris, Cat. No. 4944). Cells were pre-incubated with 5 µM PO1 in phenol-red-free DMEM for 20 min at 37°C, followed by incubation with fresh phenol-red-free DMEM containing Molidustat. PO1 was measured 90 min after the addition of Molidustat using a plate reader (Tecan Spark 20, excitation wavelength 543 nm, emission 545–750 nm). For antioxidant rescue, cells were treated with 5 mM N-acetyl-L-cysteine (NAC; Sigma-Aldrich, A7250) in addition to Molidustat. ROS levels were normalised to untreated controls.

### Synthesis of IOX4 beads

The ester group of IOX4 (Tocris Bioscience, 5084; 25 mg, 0.075 mmol) was cleaved by treatment with 90% (vol/vol) trifluoroacetic acid (TFA) in distilled water at room temperature for 2 hr, following a previously reported procedure (Chan et al., 2015). After generation of the carboxylic group, the solvent was evaporated under reduced pressure, and the resulting carboxyl-derivative of IOX4 was used directly without further purification. ω-Aminohexyl-Sepharose 4B beads, preswollen in 20% ethanol (Sigma-Aldrich, A6011; 1 ml settled gel, amine loading 8 μmol/ml), were transferred to a fritted solid-phase synthesis vessel equipped with a 20 µm polyethylene frit and washed twice with 1 ml of distilled water. Solutions containing carboxyl-derivative of IOX4 (8, 4, 2, 1, or 0.5 μmol) and N-ethyl-N′-(3-dimethylaminopropyl) carbodiimide hydrochloride (EDC; 80, 40, 20, 10, or 5 μmol, respectively) were prepared in 1 ml of distilled water, maintaining a 1:10 molar ratio of IOX4 derivative to EDC as recommended by the commercial protocol. Each solution was added to the pre-washed beads (1 ml, 8 μmol amine loading), and the suspensions were gently rotated at room temperature overnight to allow amide bond formation. As a control, ω-Aminohexyl-Sepharose 4B beads (1 ml, 8 μmol) were coupled to maleic acid (4 μmol) under identical carbodiimide-mediated coupling conditions. Following coupling, all bead samples were filtered and washed thoroughly with distilled water (≥3×). Qualitative ninhydrin and TNBS assays revealed a progressive decrease in free primary amines with increasing free-carboxy IOX4 equivalents, consistent with the expected coupling stoichiometry. Residual free amino groups on the beads were capped by treatment with acetic anhydride (80 μmol) and N,N-diisopropylethylamine (200 μmol) in 1 ml of 50% (vol/vol) dioxane in distilled water. The reaction mixtures were gently rotated at room temperature for 30 min. The beads were then filtered and washed extensively with distilled water (≥3×), and successful capping was confirmed by qualitative ninhydrin and TNBS assays, confirming the generation of IOX4-Sepharose beads (Hancock and Battersby, 1976; Gude et al., 2002).

### IOX4 assay

HT29 cells were lysed in 50 mM Tris-HCl (pH 7.4), 5% glycerol, 150 mM NaCl, 1.5 mM MgCl_2_, 0.4% NP-40, 200 µM ascorbic acid, and 10 µM FeSO_4_ with one EDTA-free protease inhibitor tablet (Roche, 11873580001) per 25 ml. Lysates were treated with benzonase (250 U/ml; Sigma-Aldrich, E1014) on ice for 1 hr, centrifuged (20,000×*g*, 1 hr, 4°C), and diluted 1:1 with detergent-free pull-down buffer. Protein concentration was adjusted to 1 mg/ml (BCA assay; Thermo Fisher Scientific, 23225). Lysates were pre-cleared with control beads (30 min, 4°C) and incubated with Molidustat (10 µM) or DMSO (30 min, 4°C). Equilibrated IOX4-Sepharose beads (35 µl) were then added and incubated for 30 min at 4°C. For analysis by SDS-PAGE and western blotting, proteins were eluted (30 min, 50°C) in 2× NuPAGE LDS sample buffer (Thermo Fisher Scientific, NP0007) containing 50 mM DTT (Sigma-Aldrich, D0632). For proteomics analysis, beads were washed twice in lysis buffer and twice in pull-down buffer, and first eluted in 2 M urea, 50 mM Tris-HCl pH 7.5 and trypsin (12.5 ng/μl) for 30 min at 27°C. The same beads were then eluted in 2 M urea, 50 mM Tris-HCl, and 1 nM DTT. The supernatants from both elutions were combined and trypsinised overnight in a humidified chamber at 37°C. The peptides were alkylated with iodoacetamide for 30 min (room temperature, in the dark), resuspended in TFA (Thermo Scientific, 28901), and desalted with C18 stagetips prior to LC-MS/MS analysis.

### Thermal proteome profiling

Cells were treated with Molidustat (10 µM, 1 hr) or DMSO prior to collection. Aliquots were heated at 37–67°C for 3 min in a thermal cycler, lysed in 1% NP-40 buffer containing protease inhibitors, and centrifuged (20,000×*g*, 20 min) to obtain soluble fractions. To remove any traces of detergent, samples were precipitated several times in ice-cold acetone (overnight, –20°C). The next day, samples were centrifuged (20,000×*g*, 20 min) and washed several times with fresh ice-cold acetone. A bath sonicator was used to ensure the pellet was fully resuspended between washes. The samples were reduced and alkylated (6 M GuHCl, 0.5 M Tris-HCl pH 8.5, 1 mg/ml chloroacetamide, 1.5 mg/ml TCEP), and digested first with LysC (Promega) for 4 hr at 37°C on a shaker (protease-to-protein ratio of 1:200), followed by 1 µg/ml mass spectrometry (MS)-grade trypsin (Promega, V5111) digestion overnight. Reactions were stopped with TFA (Thermo Scientific, 28901). Peptides were desalted on homemade C18 StageTips and resuspended in 50 mM triethylammonium bicarbonate before labelling with Tandem Mass Tags (TMT-10plex; Thermo Fisher Scientific, 90110). Peptides were desalted again on commercial C18 StageTips and fractionated off-line prior to LC-MS/MS analysis.

Thermal stability profiles were generated using the Easy50 pipeline adapted from Savitski et al., 2014. Protein abundance was normalised to the 37°C reference, and nonlinear least-squares regression was used to fit sigmoidal denaturation curves following the equation: y=1/(1+exp((Tm – x)/b)), where Tm represents the apparent melting temperature and b the slope parameter. Proteins showing reproducible >1°C shifts in Tm (p<0.05) and/or reproducible changes (Δ>0.1) in soluble abundance at 67°C (p<0.05) between treatment and control were considered compound interactors. Volcano plots were created using GraphPad Prism.

### Proteomics analyses

Cells were lysed in PAC buffer (5% SDS, 100 mM Tris-HCl pH 8.5, 1 mg/ml chloroacetamide, 1.5 mg/ml TCEP), heated at 95°C for 30 min, and sonicated. Lysates were processed for automated digestion (8 hr) on the KingFisher Duo (Thermo Fisher Scientific) using MagReSyn HILIC beads (ReSyn Biosciences) and 70% acetonitrile. Washing was performed with 95% acetonitrile and 70% ethanol, and digestion was carried out with 1 µg/ml MS-grade trypsin (Promega) in 50 mM triethylammonium bicarbonate. Reactions were stopped with trifluoroacetic acid (TFA; Thermo Scientific, 28901). Peptides were desalted on C18 StageTips and analysed on an Orbitrap Fusion Lumos Tribrid Mass Spectrometer (Thermo Fisher Scientific) coupled to an Ultimate 3000 Nano LC with a C18 packed emitter (Aurora, IonOptiks). Peptides were separated using a 40 min acetonitrile gradient followed by a 10 min 80% wash in 0.5% acetic acid. Data-independent acquisition (DIA) was performed across m/z 350–1650 at 120 k resolution, followed by MS/MS on 45 overlapping windows (200–2000 Da) at 30 k resolution (NCE 28). Data were analysed using DIA-NN (version 2.3.0 Academia) or FragPipe platform (v.23.1) against the *Homo sapiens* UniProt reference proteome. Statistical calculations, such as Student’s t-tests, ANOVA, and z-scores, were performed using Perseus software (Tyanova et al., 2016). Heatmaps and hierarchical clustering were conducted using the Morpheus online tool (https://software.broadinstitute.org/Morpheus). Volcano plots were created using the VolcaNoseR online tool (Goedhart and Luijsterburg, 2020). Gene ontology analyses were obtained using the ShinyGO 0.80 online tool (Ge et al., 2020) and The Molecular Signature Databases (MSigDB) (Aleksander et al., 2023; Ashburner et al., 2000). For proteomic synergy analyses, input intensities were log_2_-transformed and normalised by median centring. For each protein, an ordinary least-squares linear model was fitted to quantify genetic interactions between the two knockouts. The knockout representing the deviation from additivity on the log_2_ scale was used as the synergy score. The null hypothesis of no interaction was tested per protein, and p-values were adjusted across proteins using the Benjamini-Hochberg false discovery rate (FDR); unless noted otherwise, significance was defined at FDR ≤0.05. To focus on coherent genetic interactions, we reported a subset of proteins in which both single knockouts changed in the same direction relative to control (increase or decrease) by at least 0.1 log_2_ units. All analyses were performed in Python using the pandas and NumPy libraries, with statistical modelling conducted in statsmodels (ordinary least squares with HC3 heteroscedasticity-consistent standard errors) and multiple-testing correction applied using the Benjamini-Hochberg FDR procedure.

Raw datasets have been uploaded to ProteomeXchange under the identifier: PXD071906.

### Metabolomics analyses

For LC-MS metabolomics, 5×10^5^ cells per well were seeded in six-well plates and cultured for 24 hr. Metabolites were extracted using 50% methanol, 30% acetonitrile, and 20% water (vol/vol) for 1 hr at –20°C, followed by centrifugation (16,100×*g*, 10 min, 4°C). Supernatants were stored at –75°C prior to analysis. Samples were randomised and analysed on a Dionex Ultimate 3000 UHPLC (Thermo Fisher Scientific) coupled to a Q Exactive Hybrid Orbitrap MS (Thermo Fisher Scientific). Metabolites were separated by HILIC using a ZIC-pHILIC analytical column (2.1×150 mm; SeQuant-MerckMillipore) with a guard column (2.1×20 mm). Mobile phase A contained 20 mM ammonium carbonate with 0.01% ammonium hydroxide; mobile phase B was acetonitrile. A linear gradient from 95% to 5% B over 20 min was followed by re-equilibration for 7 min at 200 µl/min, 45°C, with a 5 µl injection volume. MS data were acquired in polarity-switching mode (m/z 70–900) at 70,000 FWHM resolution. Metabolites were identified by accurate mass (<5 ppm) and retention time matching to an in-house standard library. Data were processed using MetaboAnalyst 5.0, Morpheus online tool. Raw data have been deposited to MassIVE under the identifier MSV000103644.

### Materials availability

The CRISPR/Cas9 knockout HT29 cell lines generated in this study are available from the corresponding authors upon request.

## Results

### Molidustat induces cell death in colonic cell lines independently of PHD inhibition

**Figure 1.**
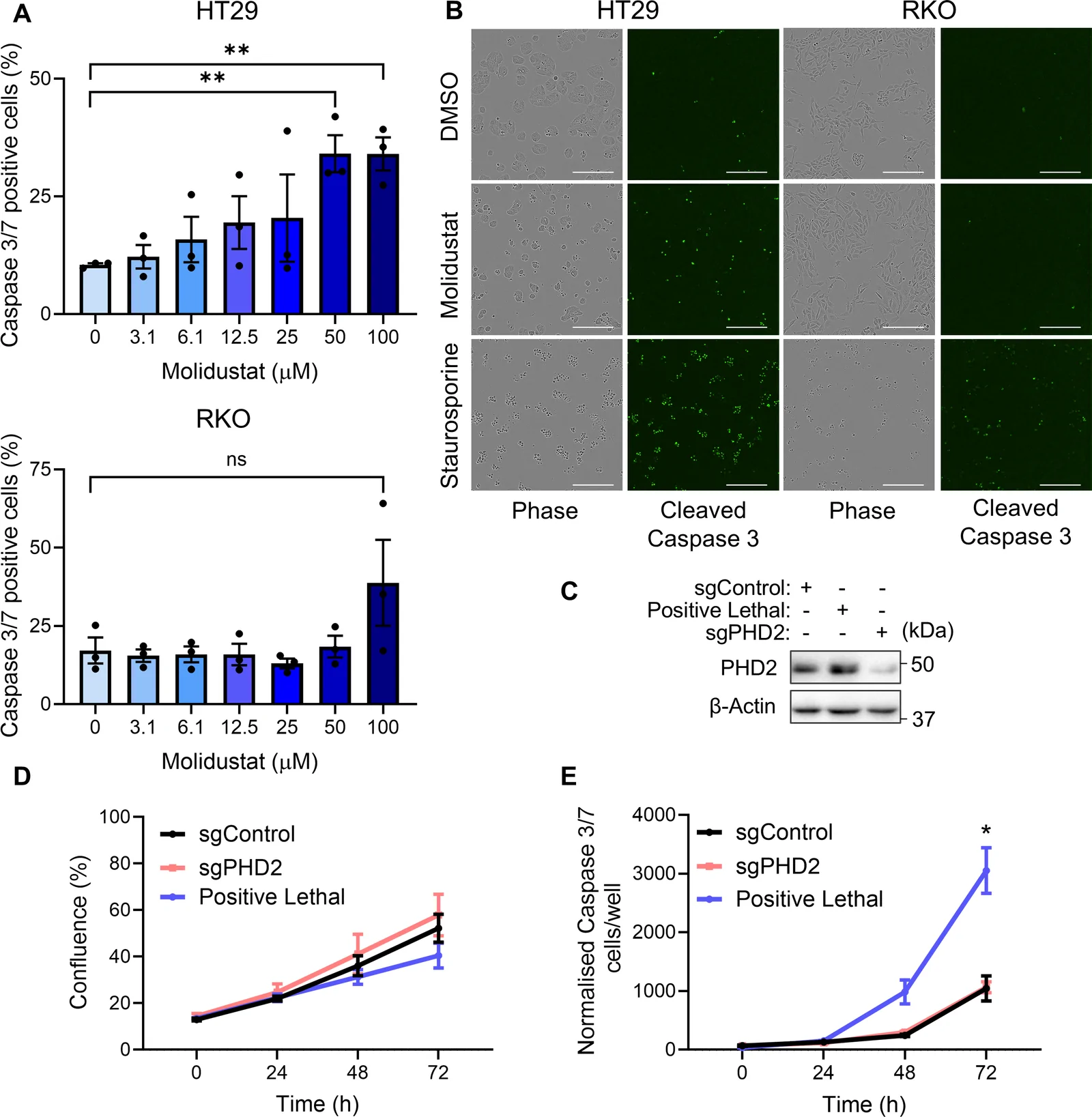
Molidustat induces cell death in colonic cell lines independently of PHD inhibition. (**A**) Percentage of cleaved Caspase-3/7 positive HT29 (top) and RKO (bottom) cells. Cells were treated with Molidustat for 48 hr at indicated concentrations; 10 μM Staurosporine was used as a positive control (100% cell death). Mean + SEM is assessed using unpaired two-tailed Student’s t-test, **p<0.01, (ns) non-significant. (**B**) Representative images of cleaved Caspase-3/7 signal in DMSO, Molidustat (90 μM), and Staurosporine-treated cells. Scale bar: 300 μm. (**C**) Representative western blot of PHD2 levels in HT29 cells. (**D**) Percentage confluency of HT29 cells post-transfection with the indicated guide RNAs. (E) Cleaved Caspase-3/7 signal in HT29 cells post-transfection with the indicated crRNAs. Mean + SEM is assessed using two-way ANOVA, *p<0.05. N=3 biologically independent experiments. Figure 1—source data 1.PDF file containing the original, uncropped western blots for Figure 1C, with the relevant bands and molecular weight markers labelled. Figure 1—source data 2.Original image files for the western blot analysis displayed in Figure 1C.

Using a substrate trap approach, we previously identified multiple components of the WNT signalling pathway as interactors of PHD hydroxylases (Rodriguez et al., 2016), suggesting that hydroxylase inhibition may modulate WNT signalling. We hypothesised that perturbing WNT signalling beyond the ‘just-right’ window required for colon cancer progression could impair proliferation and/or induce cell death, offering a potential therapeutic strategy. In murine colitis models, hydroxylase inhibitors were well tolerated and reduced inflammation (Cummins et al., 2008; Robinson et al., 2008), supporting investigation of their effects in canonical, non-inflammation-driven colon cancer. We employed two well-studied colon cancer cell lines: RKO, which lacks mutations in the WNT pathway, and HT29, which carries a canonical APC truncation (Berg et al., 2017; Ahmed et al., 2013). Cells were treated with increasing concentrations of Molidustat, a clinically approved inhibitor of PHD2, the most abundantly expressed EglN/PHD hydroxylase in these models (Berra et al., 2003; Appelhoff et al., 2004). In HT29 cells, Molidustat induced a clear dose-dependent increase in Caspase-3/7 activity, whereas RKO cells responded only at the highest concentration (Figure 1A and B). The effect appeared to be independent of HIF stabilisation as Molidustat stabilised HIF1α and induced the expression of its downstream targets, to a similar extent in both cell lines (Figure 1—figure supplement 1A and B). To determine whether PHD2 inhibition accounted for this effect, we generated PHD2 knockout HT29 cells using CRISPR-Cas9 (Figure 1C). Surprisingly, PHD2 loss neither reduced proliferation nor induced Caspase-3/7 activity (Figure 1D and E). Thus, although Molidustat is characterised as a PHD2 inhibitor, its pro-apoptotic effects cannot be explained by PHD2 inhibition alone. As Molidustat may interact with additional proteins not affected by PHD2 knockout, we hypothesised that the compound exerts the pro-apoptotic function via other targets.

**Figure 1—figure supplement 1.**
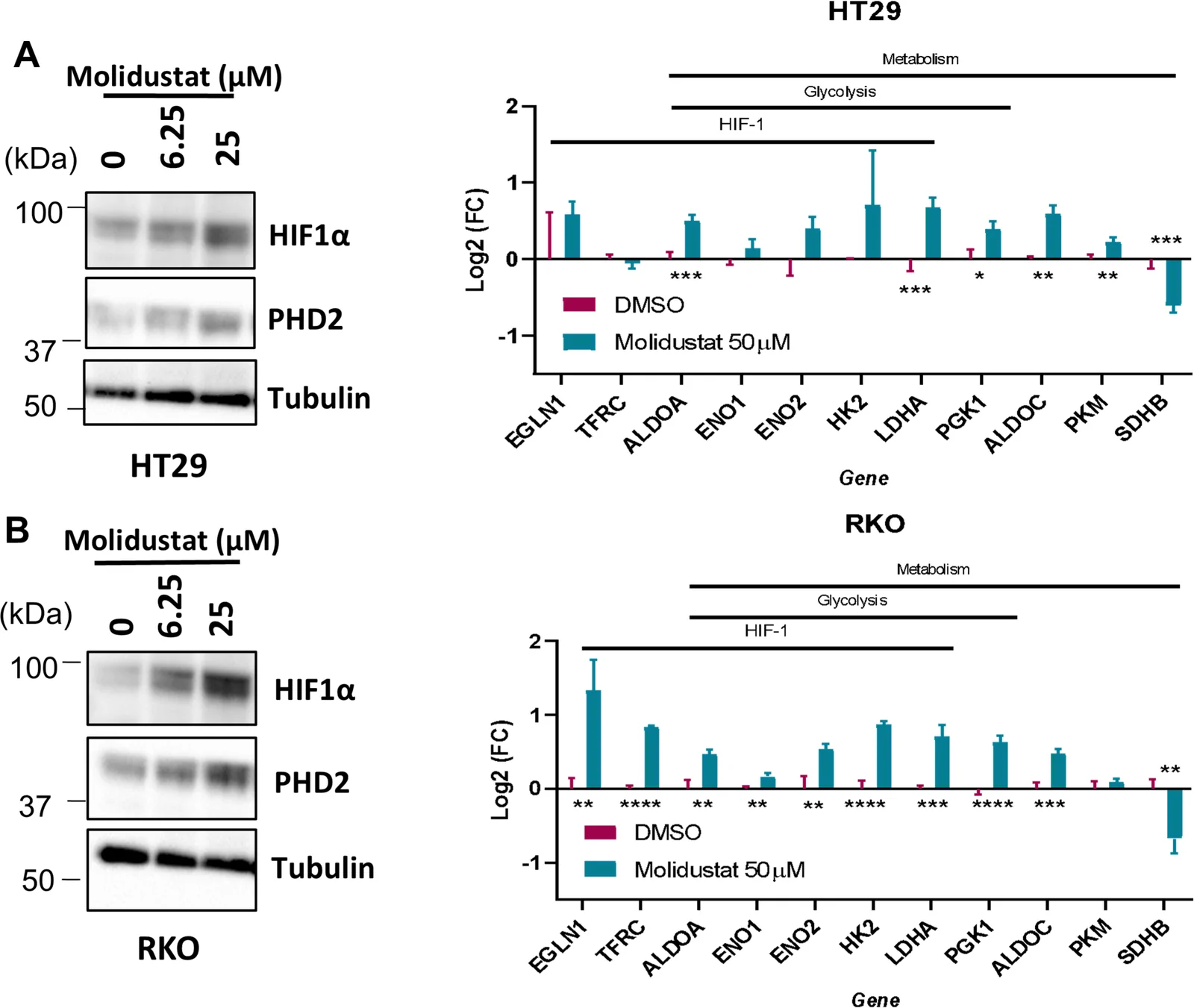
Molidustat induces HIF signalling in colonic cell lines. (**A**) HT29 cells treated with Molidustat for 16 hr. Left: Representative western blot of HIF1α and PHD2 at the indicated Molidustat concentrations (0, 6.25, 25 μM), with β-tubulin as a loading control. Right: Protein levels of HIF-responsive genes after treatment with 50 μM Molidustat, expressed as log_2_ fold change relative to DMSO. (B) As in A, for RKO cells. Mean + SEM is assessed using Student’s t-test, *p<0.05, **p<0.01, ***p<0.001, ****p<0.0001. N=3 biologically independent experiments. Figure 1—figure supplement 1—source data 1.PDF file containing the original, uncropped western blots for Figure 1—figure supplement 1A and B, with the relevant bands and molecular weight markers labelled. Figure 1—figure supplement 1—source data 2.Original image files for the western blot analyses displayed in Figure 1—figure supplement 1A and B.

### TPP reveals multiple Molidustat targets

**Figure 2.**
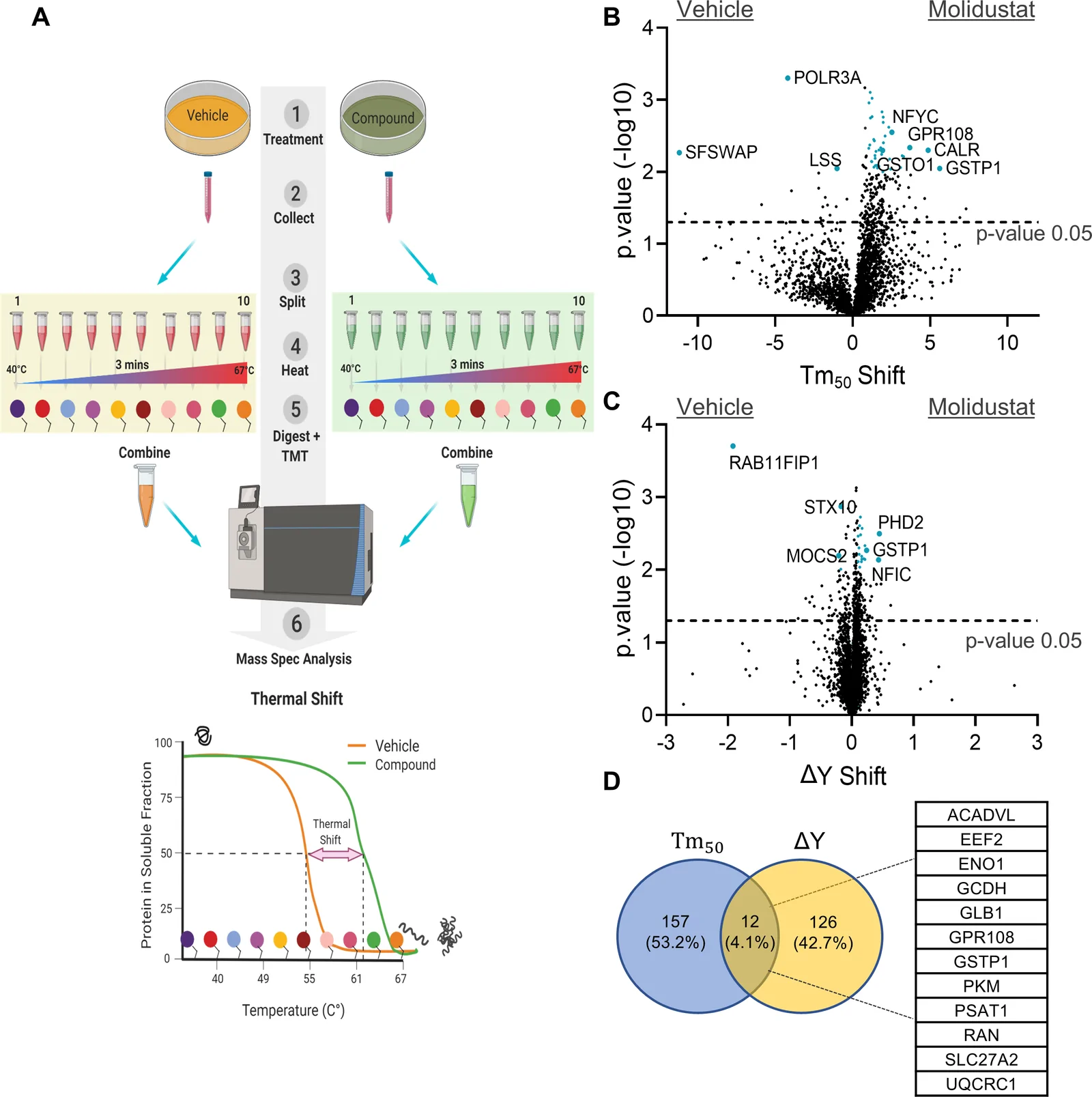
Thermal proteome profiling reveals multiple Molidustat targets. (**A**) Intact cells are treated with compound or vehicle control (1). Cells are collected (2), split into fractions (3) with each fraction heated to a different temperature (4). After heat treatment, cells are lysed and proteins are digested to peptides (5). The peptides in each fraction are labelled with a unique TMT tag, which can be analysed using mass spectrometer (6), generating melting curves and Tm50 values for the whole proteome. (**B**) Volcano plot of hits identified using the Tm50 method. Tm50 shift and –log_10_ p-value are shown. Proteins exhibiting a significant shift (<0.05) greater than 1°C were considered potential targets. (**C**) Volcano plot of all hits identified using the ΔY method. ΔY shift and –log_10_ p-value are shown. Proteins with a significant increase (<0.05) in ΔY greater than 0.1 were taken forward as potential hits. (**D**) Venn diagram showing the overlap of targets identified by both methods. Twelve proteins identified by both methods are shown on the right. N=3 biologically independent experiments. Panel A was created using BioRender.com.

To identify proteome-wide targets of Molidustat, we performed thermal proteome profiling (TPP) (Franken et al., 2015). TPP determines the temperature-dependent denaturation profiles of proteins across the proteome (Figure 2A), enabling systematic identification of putative drug-protein interactions. HT29 cells were treated in triplicate with Molidustat or DMSO for 1 hr, aliquoted, heated between 40°C and 67°C, lysed, and fractionated to separate denatured from soluble proteins. Soluble fractions were processed and analysed by LC-MS/MS. Melting curves were normalised using empirically defined thermostable proteins and analysed using two independent algorithms. Proteins exhibiting a thermal shift >1°C (ΔTm50) and/or a significant change in soluble abundance at 67°C (ΔY) were classified as hits (Figure 2B and C). A total of 295 proteins met these criteria, including PHD2 (Supplementary file 1). Consistent with previous reports, Molidustat did not alter the stability of other dioxygenases such as PLOD1 (Figure 2—figure supplement 1A–D). Twelve proteins were identified by both algorithms (Figure 2D). However, altered thermal stability can arise either from direct Molidustat binding or from Molidustat-induced post-translational modifications affecting protein stability (Le Sueur et al., 2022). Additional methods are therefore required to distinguish true interactors from indirect effects. Overall, the observed thermal shifts indicate that Molidustat perturbs the stability of a broad set of proteins, consistent with engagement of multiple cellular targets.

**Figure 2—figure supplement 1.**
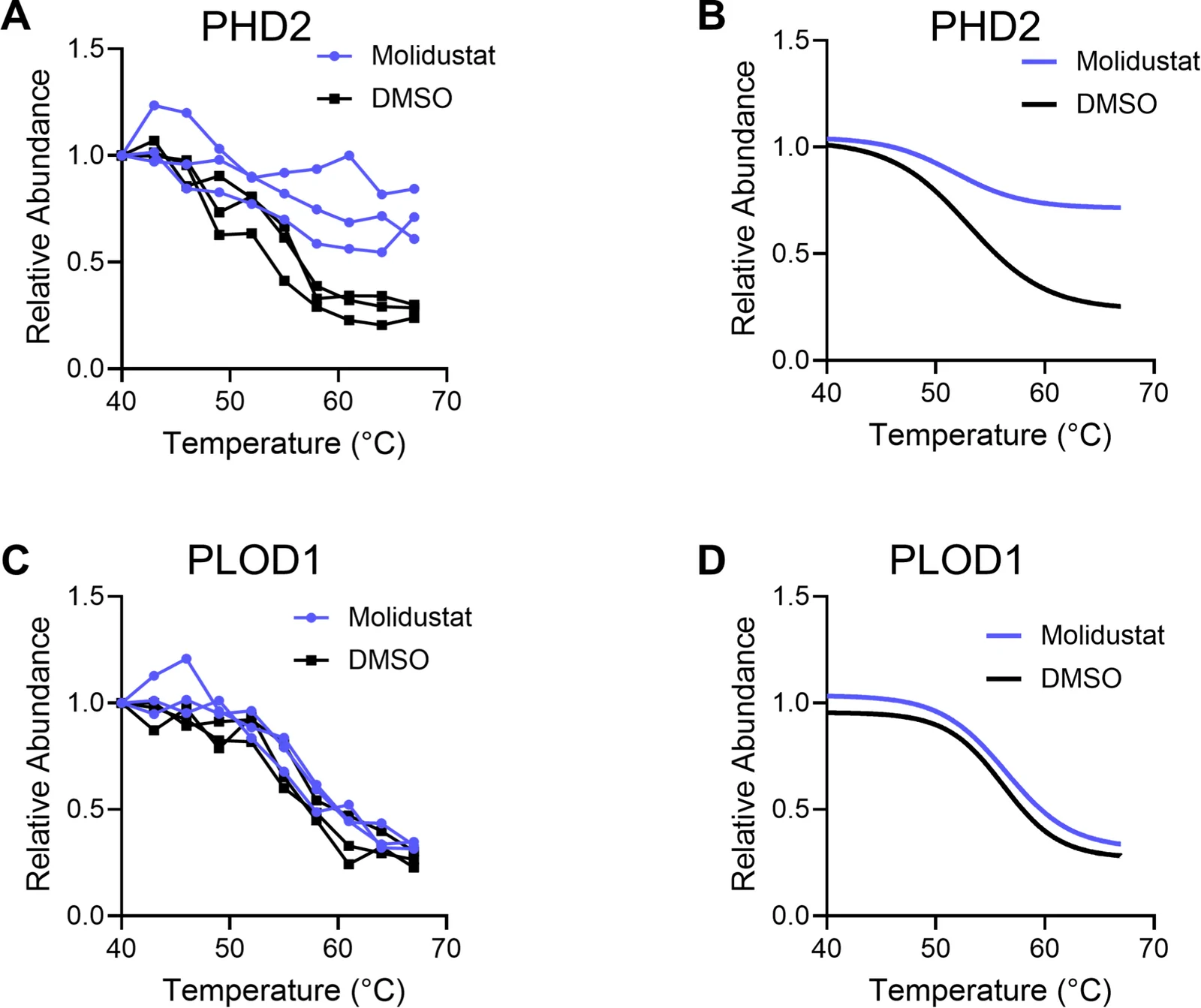
Molidustat increases PHD2 thermal stability. (**A**) Raw values for PHD2 in samples treated with Molidustat (blue) or DMSO (black). (**B**) Sigmoidal curve fits for PHD2 in samples treated with Molidustat (blue) or DMSO (black). (**C**) Raw values for PLOD1 in samples treated with Molidustat (blue) or DMSO (black). (**D**) Sigmoidal curve fits for PLOD1 in samples treated with Molidustat (blue) or DMSO (black). N=3 biologically independent experiments.

### Molidustat inhibits GSTP1 activity

**Figure 3.**
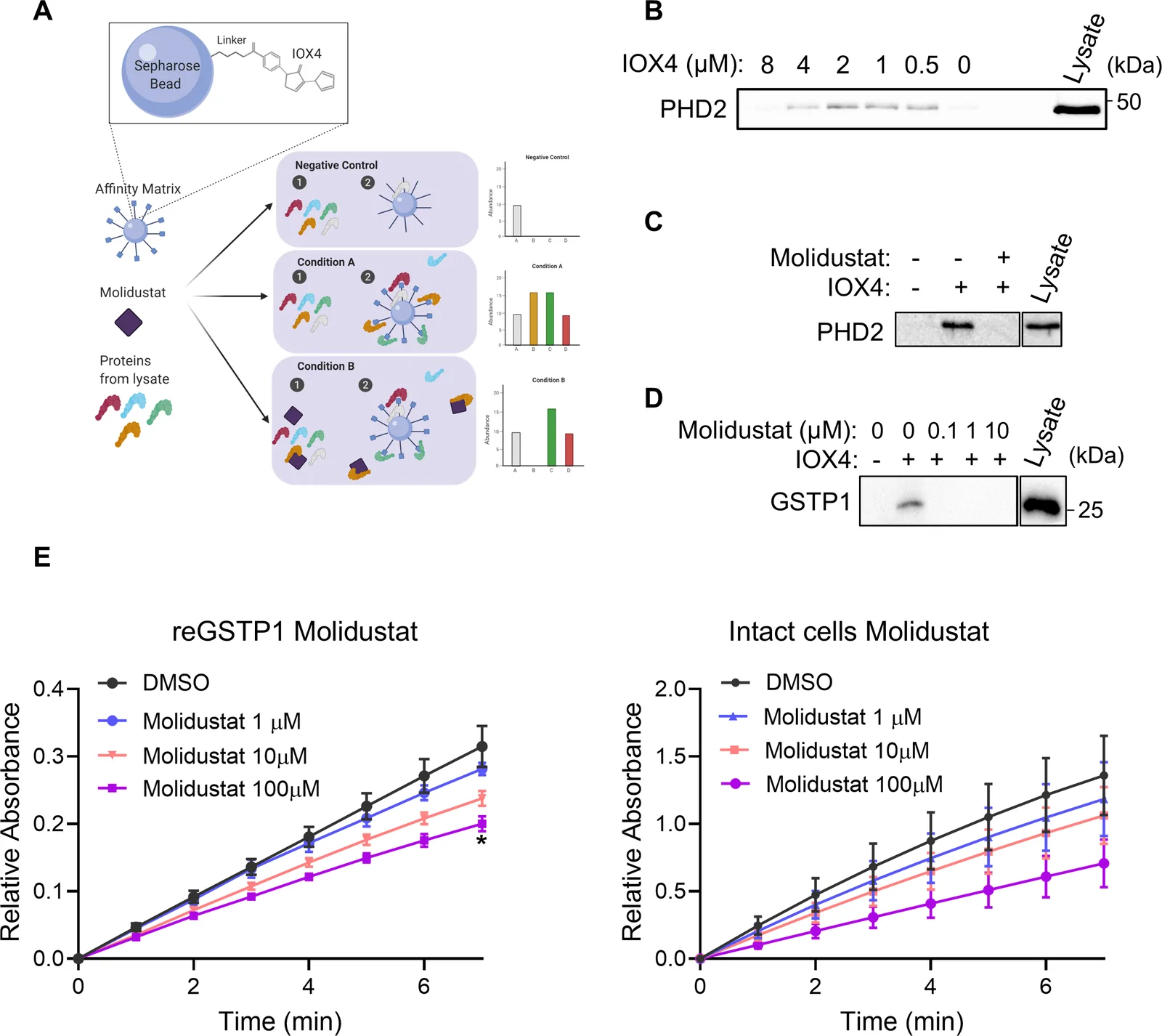
Molidustat inhibits GSTP1 activity. (**A**) ω-Aminohexyl sepharose beads were coupled with IOX4. Cell lysate is washed over empty beads (negative controls), IOX4 coupled beads (condition A), or IOX4 coupled beads after incubation with Molidustat (condition B). Samples are analysed by western blot or mass spectrometry. (**B**) Western blot of PHD2 pull-down with IOX4-conjugated beads across a concentration range (0.5–8 μM). (**C**) Competition assay with Molidustat. Lysates were pre-incubated with Molidustat prior to incubation with IOX4-conjugated beads, PHD2 pull-down was assessed using western blot. (**D**) Western blot of GSTP1 binding to IOX4 beads with or without prior incubation with Molidustat. (E) Recombinant (left) or cell-based (right) GSTP1 activity assay with Molidustat (1, 10, 100 μM) or DMSO; time-course of CDNB-GSH conjugation monitored at 340 nm and plotted as relative absorbance (arbitrary units, AU). Mean + SEM is assessed using two-way ANOVA, *p<0.05, N=3 biologically independent experiments. Panel A was created using BioRender.com. Figure 3—source data 1.PDF file containing the original, uncropped western blots for Figure 3B, C, and D, with the relevant bands and molecular weight markers labelled. Figure 3—source data 2.Original image files for the western blot analyses displayed in Figure 3B, C, and D.

To refine target identification, we implemented an orthogonal chemical proteomic competition assay resembling an affinity pull-down (Joberty et al., 2016; Figure 3A). Protein lysates were incubated with an immobilised compound or control matrix in the presence or absence of Molidustat. In the absence of free drug, interacting proteins co-precipitated; in its presence, binding sites were blocked, reducing their recovery. Comparing precipitated proteomes with and without Molidustat pre-treatment enabled the identification of specific binders. We generated the affinity matrix by coupling a close analogue of Molidustat IOX4 (Yeh et al., 2017) to agarose beads and optimised coupling conditions using the characterised IOX4-PHD2 interaction (Figure 3B and C). MS analyses identified multiple proteins altered where pre-treatment with Molidustat reduced the affinity to the IOX4 beads (Supplementary file 2). Of those, GSTP1 showed the most striking changes in the precipitation levels and western blot analyses confirmed that pre-incubation with Molidustat prevented GSTP1 binding to IOX4 beads (Figure 3D, Figure 3—figure supplement 1A). GSTP1 also appeared as a hit in the TPP dataset (Figure 2B and C), supporting its classification as a bona fide Molidustat interactor. Consistent with inhibition of GSTP1, a key cellular detoxification enzyme, Molidustat treatment led to a significant increase in intracellular ROS levels. Co-treatment with the antioxidant NAC, a ROS scavenger (Pedre et al., 2021), abolished the Molidustat-induced ROS increase, supporting a functional link between GSTP1 inhibition and redox imbalance (Figure 3—figure supplement 1B). To further explore the molecular basis of GSTP1 binding, we performed in silico docking analyses of Molidustat (Morgan et al., 1996; Prade et al., 1997; Flatgaard et al., 1993; Zhang et al., 2021), which indicated a potential binding site spanning the canonical GST-binding and hydrophobic pocket, which could prevent substrate/GST binding (Figure 3—figure supplement 1C). To test whether binding of Molidustat to GSTP1 inhibited GST activity, we measured GSTP1 activity using recombinant GSTP1 and the synthetic substrate CDNB (Habig and Jakoby, 1981). Molidustat inhibited GSTP1 activity in a dose-dependent manner (Figure 3E), comparable to inhibition by EA, another known GSTP1 inhibitor (Allocati et al., 2018; Figure 3—figure supplement 1D). To assess intracellular activity, we repeated the assay in intact HT29 cells and observed reduced GSTP1 activity dose-dependently, whereas EA had no detectable effect (Figure 3E; Figure 3—figure supplement 1D). Given the observed increase in ROS, we tested whether pharmacological inhibition of GSTP1 could recapitulate the Molidustat-induced apoptosis in PHD2 knockout cells using Ezatiostat. However, no significant differences in Caspase-3/7 signal were observed between WT cells and GSTP1 knockout controls (Figure 3—figure supplement 1E), rendering these results inconclusive. Taken together, our chemical proteomic, biochemical, and structural analyses identify GSTP1 as a prominent off-target of Molidustat and demonstrate direct inhibition of its enzymatic activity.

**Figure 3—figure supplement 1.**
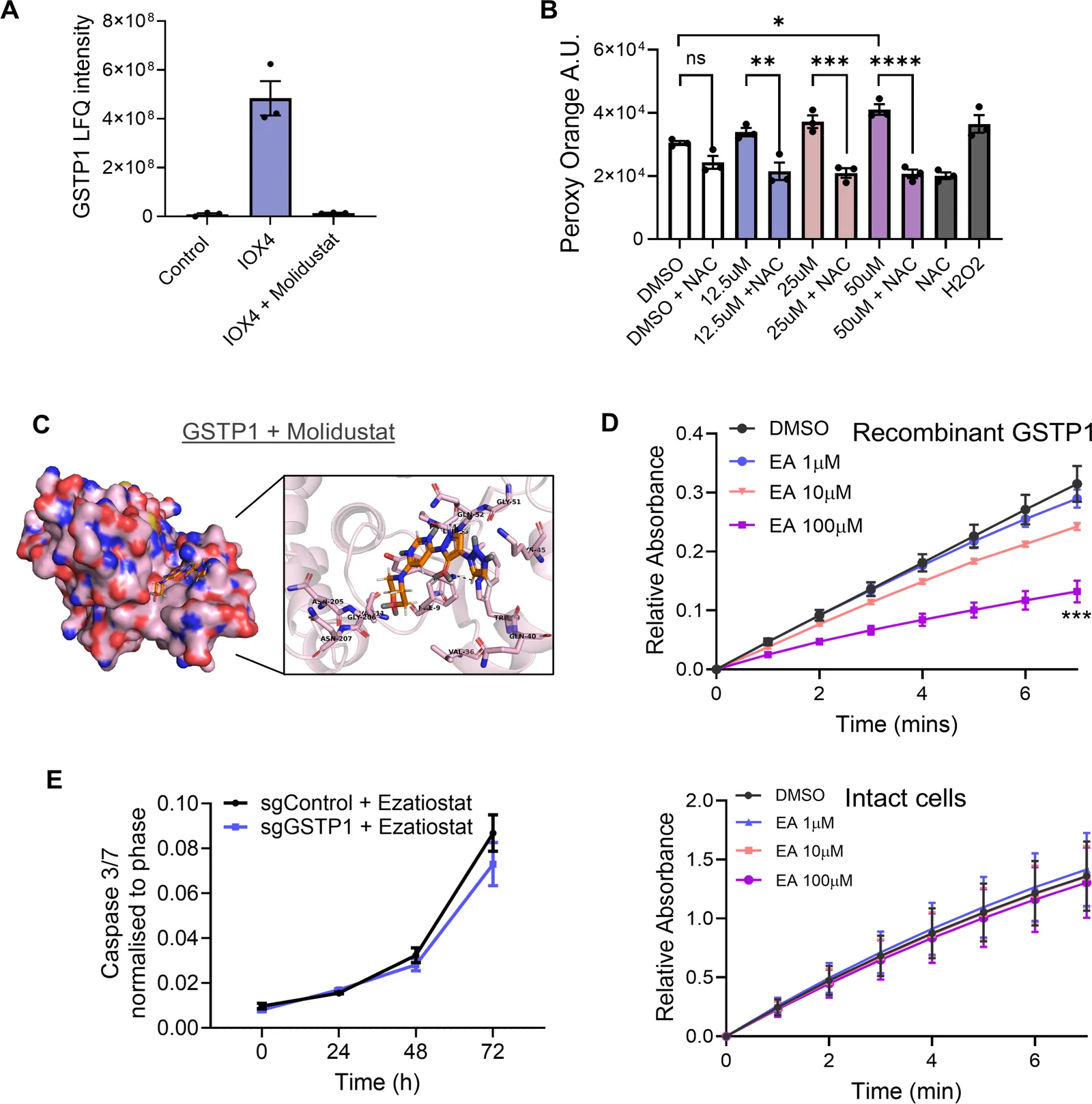
Molidustat inhibits GSTP1 activity. (**A**) Mass spectrometry (MS) analyses of GSTP1 levels in the indicated conditions. Protein intensity is shown as label-free quantitation (LFQ) values. (**B**) HT29 cells were treated with increasing concentrations of Molidustat. Reactive oxygen species (ROS) were measured using Peroxy Orange 1 (PO1) and expressed as arbitrary units (AU). At 50 μM Molidustat, a significant increase in PO1 fluorescence was observed. Co-treatment with 5 mM N-acetyl-L-cysteine (NAC), a ROS scavenger, abolished the Molidustat-induced signal. Mean + SEM is assessed using one-way ANOVA, *p<0.05, **p<0.01, ***p<0.001, ****p<0.0001, (ns) non-significant. N=3 biologically independent experiments. (**C**) Molecular docking of Molidustat to GSTP1 protein. Residues in the active site are highlighted on the right. (**D**) Recombinant (left) or cell-based (right) GSTP1 assay with ethacrynic acid (EA) or DMSO; time-course of CDNB-GSH conjugation monitored at 340 nm and plotted as relative absorbance (arbitrary units, AU). (**E**) Cleaved Caspase-3/7 signal curve in the indicated cell lines treated with Ezatiostat. Mean + SEM is assessed using two-way ANOVA. N=1 biologically independent experiment.

### Combined PHD2 and GSTP1 loss drives synergistic proteomic changes associated with apoptosis

**Figure 4.**
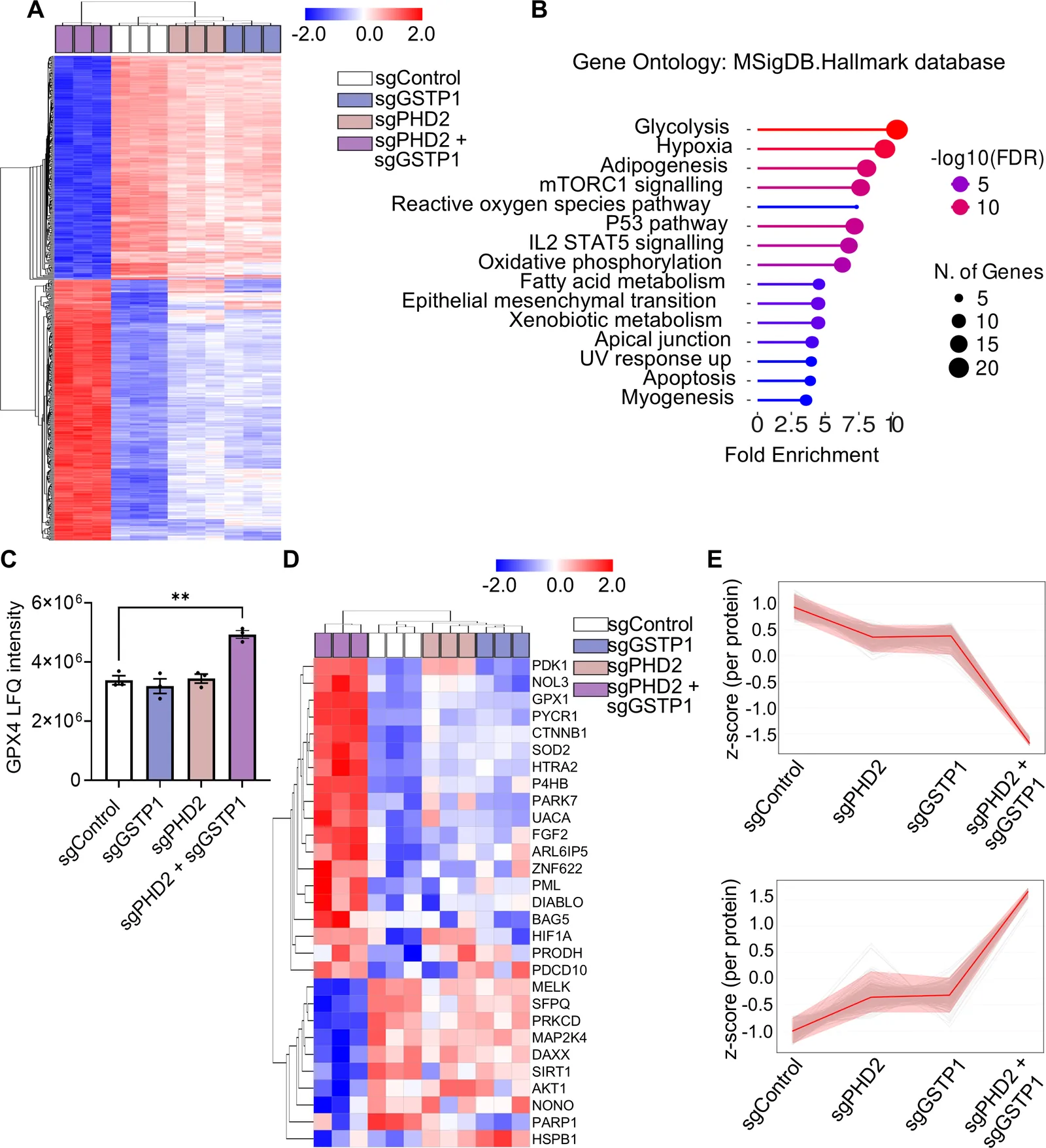
Combined PHD2 and GSTP1 loss drives synergistic proteomic changes associated with apoptosis. (**A**) Heatmap of top 500 variable proteins in the tested dataset. The p-values were calculated using one-way ANOVA. Hierarchical clustering of columns (samples) and rows (proteins detected) was performed using Euclidean distance. (**B**) Protein enrichment analysis of bottom cluster using MSigDB.Hallmark database as a reference. (**C**) Mass spectrometry (MS) analyses of GPX4 levels in the cell populations. GPX4 intensity is shown as a label-free quantitation (LFQ) values. Mean + SEM is assessed using unpaired two-tailed Student’s t-test, **p<0.01. (**D**) Heatmap of GO term ‘Intrinsic Apoptotic Signalling Pathway in Response to Oxidative Stress’ proteins found in the tested dataset. Hierarchical clustering of columns (samples) and rows (proteins detected) was performed using Euclidean distance. (**E**) Synergy profiles of downregulated (top) and upregulated (bottom) proteins, expressed as per-protein z-scores. A total of 361 downregulated (N=361) and 314 upregulated (N=314) proteins exhibited synergistic behaviour.

To test whether genetic ablation of GSTP1 and PHD2 recapitulated the apoptotic phenotype induced by Molidustat (Figure 1A and B), we generated single and double knockout HT29 cell lines and profiled them using quantitative proteomics (Supplementary file 3). LFQ analysis confirmed efficient loss of GSTP1 and PHD2, and HIF1α accumulation in PHD2-deficient backgrounds (Figure 4—figure supplement 1A and B). Unsupervised clustering of the top 500 ANOVA-variable proteins identified two major expression clusters, with the most pronounced alterations occurring in the double knockout (Figure 4A). Notably, loss of either PHD2 or GSTP1 alone modulated a shared subset of proteins in similar directions, and the combined knockout produced an amplified response, resulting in potentiation of both upregulated and downregulated protein cohorts. The downregulated cluster was enriched for cell-cycle-associated Hallmarks, suggesting reduced proliferative capacity, whereas the upregulated module was enriched for glycolysis, ROS pathway, and apoptosis (Figure 4B, Figure 4—figure supplement 1C). Within the upregulated proteins, GPX4, a key GSH-dependent lipid peroxide reductase (Seiler et al., 2008), was elevated (Figure 4C), consistent with increased lipid oxidative pressure. This prompted us to examine proteins annotated to the GO term ‘Intrinsic Apoptotic Signalling Pathway in Response to Oxidative Stress’ (Aleksander et al., 2023; Ashburner et al., 2000). These were segregated into two coordinated clusters that distinguished the double knockout from all other genotypes (Figure 4D). Mitochondrial apoptosis effectors DIABLO and HTRA2, the oxidative-stress-responsive tumour suppressor PML, and SOD2 were upregulated. In contrast, pro-survival factors, including AKT1, were downregulated, further supporting an apoptosis-permissive, oxidative-stress-associated state specific to the double knockout (Figure 4D). To formally assess genetic interaction effects, we computed per-protein synergy scores across all genotypes. This analysis revealed widespread synergistic behaviour in the double knockout, with 361 downregulated and 314 upregulated proteins exhibiting non-additive responses (Figure 4E). Synergistically altered proteins were highly enriched for cell-cycle suppression and apoptotic signalling (Figure 4—figure supplement 1D and E), indicating that combined loss of PHD2 and GSTP1 drives a coordinated, systems-level shift towards growth arrest and cell death that exceeds the effects of either perturbation alone, with potentially lethal consequences for cellular viability.

**Figure 4—figure supplement 1.**
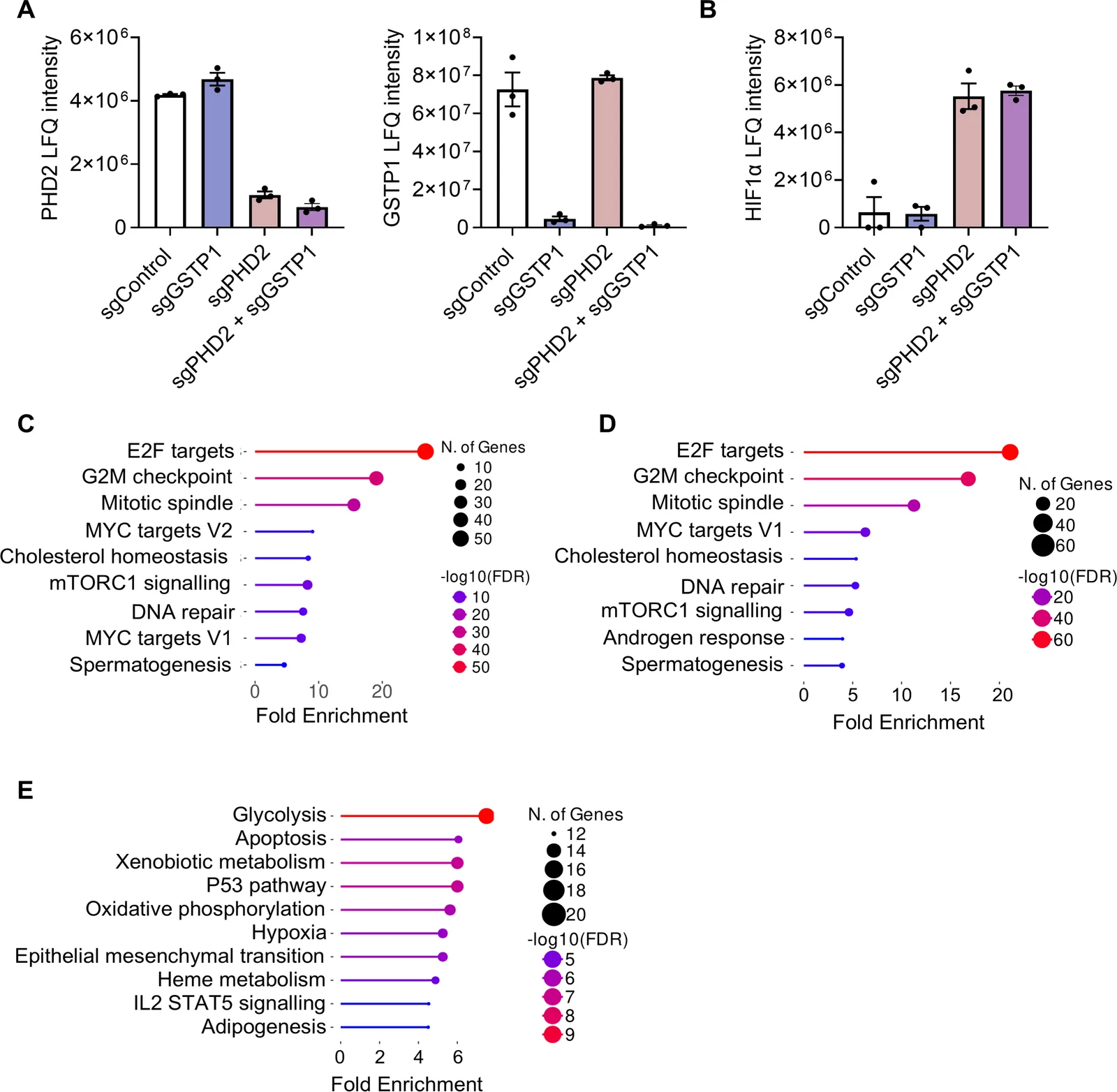
Proteomics analyses of HT29 knockout cell models. (**A**) Mass spectrometry (MS) analyses of PHD2 (left) or GSTP1 (right) levels in the cell populations. Protein intensity is shown as label-free quantitation (LFQ) values. (**B**) MS analyses of HIF1α levels in the cell populations. HIF1α intensity is shown as label-free quantitation (LFQ) values. (**C**) Protein enrichment analysis of the top cluster from Figure 4A using the MSigDB.Hallmark database as a reference. (**D**) Protein enrichment analysis of synergistic, downregulated proteins (as seen in Figure 4E) using the MSigDB.Hallmark database as a reference. (**E**) Protein enrichment analysis of synergistic, upregulated proteins (as seen in Figure 4E) using the MSigDB.Hallmark database as a reference. N=3 biologically independent experiments.

### Metabolic imbalance and apoptotic vulnerability following combined PHD2 and GSTP1 loss

**Figure 5.**
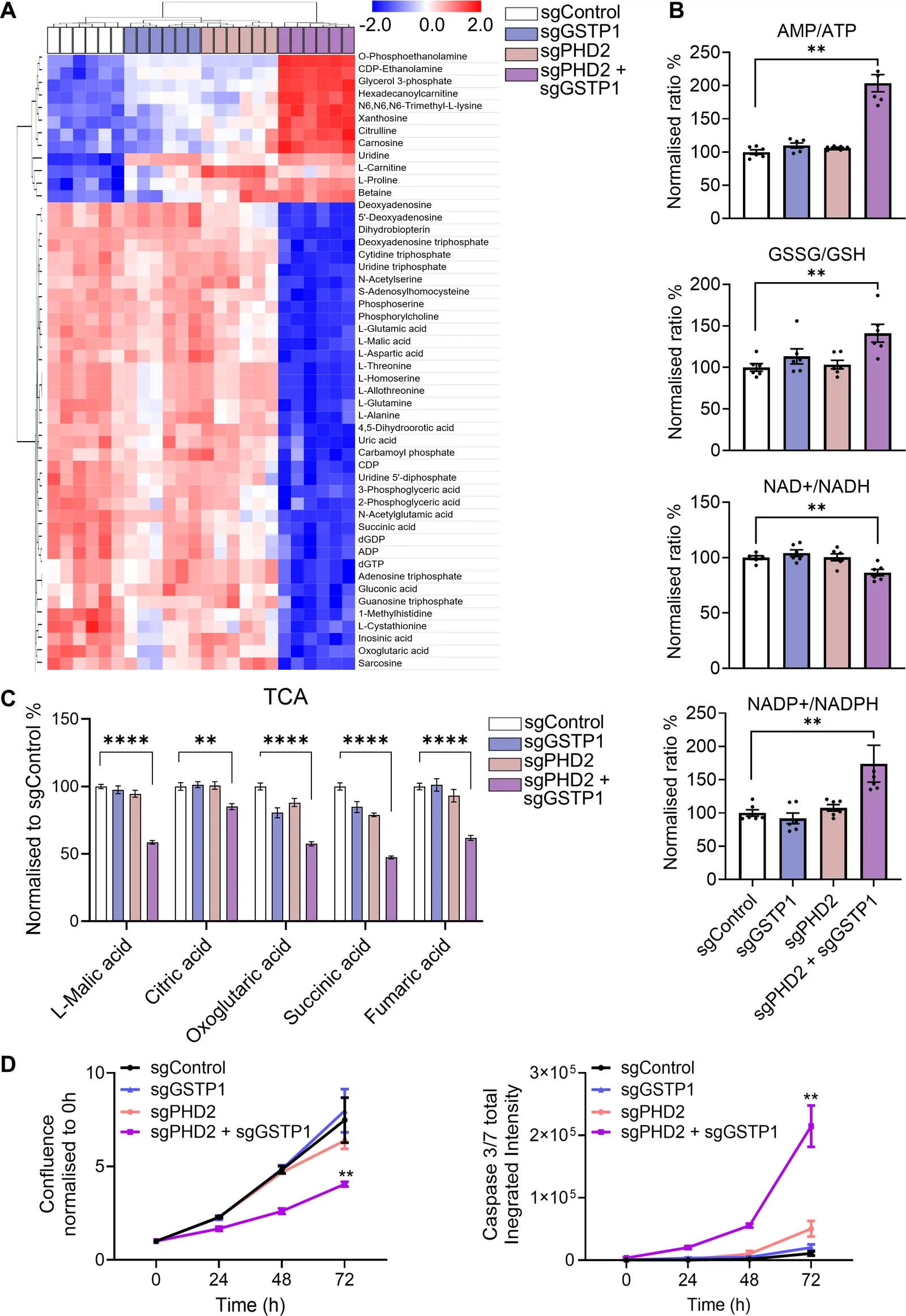
Metabolic imbalance and apoptotic vulnerability following combined PHD2 and GSTP1 loss. (**A**) Heatmap of top 50 variable metabolites in the tested dataset. The p-values were calculated using one-way ANOVA. Hierarchical clustering of columns (samples) and rows (metabolites detected) was performed using Euclidean distance. (**B**) Metabolomics analyses of AMP/ATP, GSSH/GSH, NAD^+^/NADH, and NADP/NADH ratios, in the indicated cell lines. Mean + SEM is assessed using unpaired two-tailed Student’s t-test, **p<0.01. (**C**) TCA cycle metabolite levels, normalised to sgControl. Mean + SEM is assessed using unpaired two-tailed Student’s t-test, **p<0.01, ****p<0.0001. (**D**) Left: Confluence curve normalised to timepoint 0 hr in the indicated cell lines. Right: Representative cleaved Caspase-3/7 signal curve in the indicated cell lines. Mean + SEM is assessed using two-way ANOVA, **p<0.01. N=3 biologically independent experiments.

To assess whether the metabolic signatures inferred from proteomic analyses were reflected at the metabolite level, we performed untargeted metabolomics across all four genotypes (Supplementary file 4). Unsupervised hierarchical clustering of ANOVA-variable metabolites revealed clear genotype-dependent separation, with the sgPHD2 and sgGSTP1 double knockout forming a distinct metabolic cluster (Figure 5A). Notably, single knockouts showed partially overlapping profiles, whereas the combined perturbation produced a qualitatively distinct metabolic state, mirroring the non-additive pattern observed at the proteome level. Quantitative analysis of key energetic and redox parameters demonstrated a pronounced imbalance in the double knockout, including an elevated AMP/ATP ratio together with increased GSSG/GSH and altered NAD^+^/NADH and NADP^+^/NADPH ratios (Figure 5B). These changes were accompanied by coordinated depletion of TCA cycle intermediates (Figure 5C), consistent with impaired mitochondrial metabolic capacity and altered redox buffering. Joint enrichment analysis of metabolomics and proteomics data further highlighted altered central carbon and amino acid metabolism in the double knockout genotype (Figure 5—figure supplement 1A). Together, these metabolic alterations point to a state of energetic stress and compromised mitochondrial function in the sgPHD2 and sgGSTP1 double knockout, with secondary effects on cellular redox balance. Consistent with this metabolic state, live-cell imaging of cleaved Caspase-3/7 revealed reduced proliferation with a pronounced increase in apoptotic signalling in the double knockout cell line (Figure 5D, Figure 5—figure supplement 1B). Notably, this apoptotic response closely resembles the phenotype induced by Molidustat treatment (Figure 1A and B). Collectively, these data indicate that combined loss of PHD2 and GSTP1 precipitates a metabolic and apoptotic collapse consistent with a synthetic lethal interaction.

**Figure 5—figure supplement 1.**
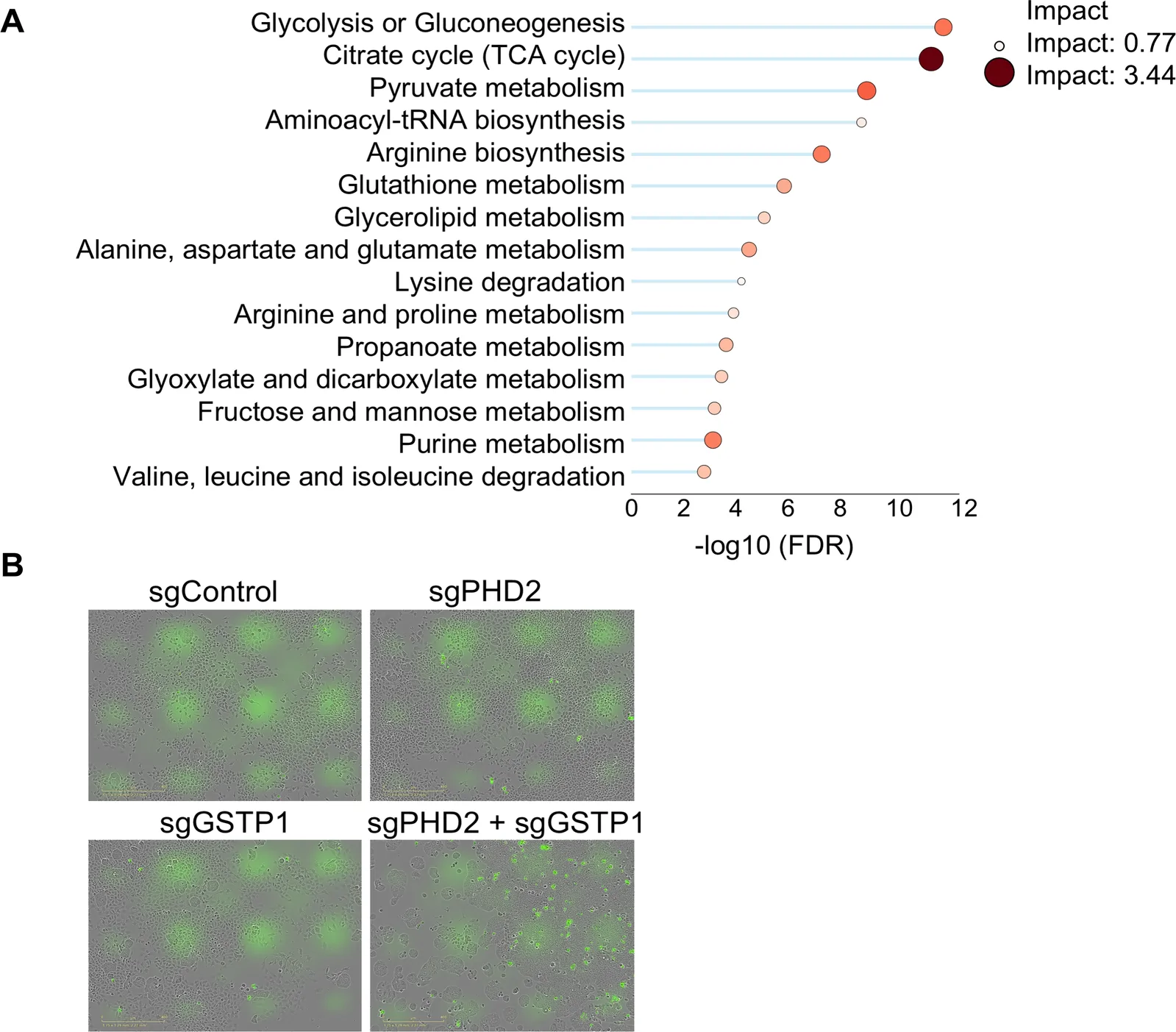
Metabolomics analyses of HT29 knockout cell models. (**A**) Enrichment analysis of metabolomics and proteomics data, showing the top 15 enriched metabolic processes in the sgPHD2 and sgGSTP1 cell line compared to sgControl. (**B**) Representative merged images of phase and cleaved Caspase-3/7 signal in the indicated cell lines. Scale bar: 400 μm. N=3 biologically independent experiments.

### Organoids display genotype-specific sensitivity to Molidustat

It has previously been reported that Molidustat treatment reduces tumour growth in an in vivo xenograft model of breast cancer (Kachamakova-Trojanowska et al., 2020). In our CRC in vivo model, we detected elevated renal *Epo* levels, indicating at least partial PHD2 inhibition in the kidney (Firmke et al., 2025; Figure 6—figure supplement 1A). However, Molidustat treatment did not affect survival or tumour characteristics (Figure 6—figure supplement 1B–F). One potential explanation is insufficient compound delivery to the colon, which we were unable to directly assess.

**Figure 6.**
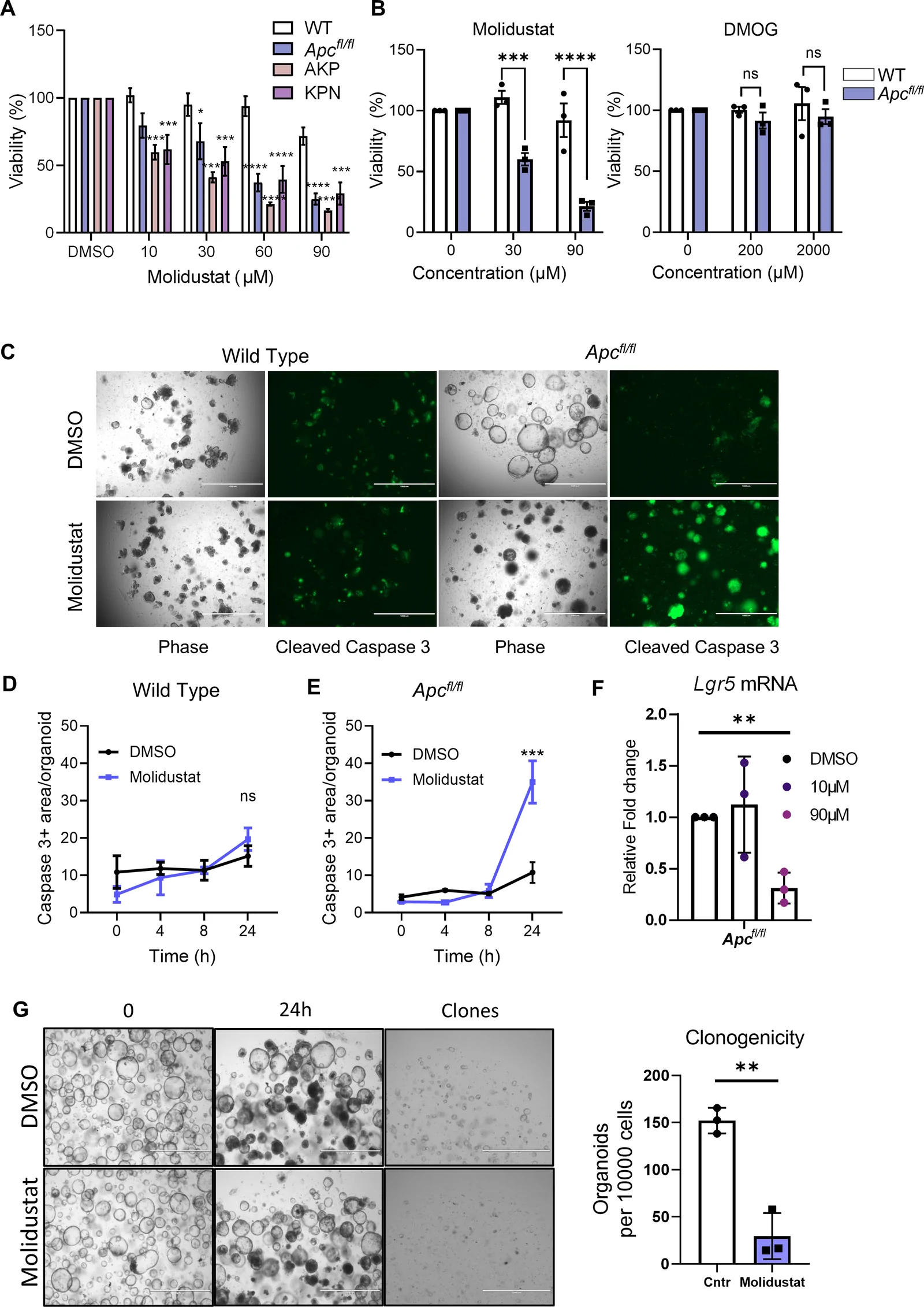
Organoids display genotype-specific sensitivity to Molidustat. (**A**) Wild-type (WT), *Apc^fl/fl^*(CMS2), AKP (CMS3), and KPN (CMS4) organoids were treated with increasing concentrations of Molidustat for 48 hr. Viability percentage was assessed using an MTT assay. Mean + SEM is assessed using two-way ANOVA using Dunnett’s correction for multiple comparisons, *p<0.05, ***p<0.001, ****p<0.0001. (**B**) 2-day-old WT or *Apc^fl/fl^* organoids were treated for 48 hr with the indicated PHD inhibitors. Organoid viability percentage was assessed using an MTT assay. Mean + SEM is assessed using unpaired two-tailed Student’s t-test, ***p<0.001, ****p<0.0001, (ns) non-significant. (**C**) Representative images of cleaved Caspase-3/7 signal in WT and *Apc^fl/fl^* organoids treated with DMSO or Molidustat at 24 hr timepoint. Scale bar: 1000 μm. (**D–E**) Quantification of cleaved Caspase-3/7 signal area in organoids at the indicated timepoints. Mean + SEM is assessed using unpaired two-tailed Student’s t-test, ***p<0.001, (ns) non-significant. N=3 biologically independent experiments. (**F**) Normalised real-time quantitative polymerase chain reaction (RT-qPCR) quantification of *Lgr5* mRNA expressed in *Apc^fl/fl^* organoids treated with DMSO or Molidustat at 24 hr timepoint two-tailed Student’s t-test, **p<0.01. N=3. (**G**) Representative images of *Apc^fl/fl^* organoids treated with DMSO or Molidustat at two timepoints and clones grown from organoids treated for 24 hr with DMSO or Molidustat. Scale bar: 1000 μm. Quantification of clones in on the right, two-tailed Student’s t-test, **p<0.01, (ns). N=3.

Given the lack of efficacy in an in vivo model, we decided to evaluate the effects of Molidustat in a more relevant system where drug delivery could be better controlled. We therefore employed murine organoids, a stem-cell-derived system that recapitulates epithelial organisation and cellular heterogeneity and enables reliable compound exposure (Ma et al., 2024). To determine whether sensitivity to Molidustat was associated with particular CRC subtypes, we included organoids derived from Genetically Engineered Mouse Models (GEMMs) representing three CMS classifications: *Apc^fl/fl^* (CMS2), *Apc*/*Kras*/*p53* (AKP) (CMS3), *Kras*/*p53*/*Notch* (KPN) (CMS4), in addition to WT controls. Molidustat moderately reduced viability in WT organoids, even at high concentrations, whereas *Apc^fl/fl^*, AKP, and KPN organoids displayed a clear dose-dependent reduction in viability (Figure 6A). These data suggest that organoids with activated WNT signalling and/or KRAS mutations are sensitive to Molidustat. To assess whether hydroxylase inhibition alone was sufficient to account for this effect, WT and *Apc^fl/fl^* organoids were treated with Molidustat or three structurally distinct hydroxylase inhibitors DMOG, JNJ-42041935, and DFO (Moreton et al., 2023; Duscher et al., 2017). DMOG, a pan-hydroxylase inhibitor, did not affect viability even at 2 mM, whereas JNJ and DFO reduced viability only at high concentrations and did so indiscriminately across genotypes, including WT organoids (Figure 6B; Figure 6—figure supplement 1G). In contrast, Molidustat selectively reduced viability in *Apc^fl/fl^* organoids (Figure 6B, Figure 6—figure supplement 1H), demonstrating that hydroxylase inhibition alone does not recapitulate the selective sensitivity observed with Molidustat. To determine whether Molidustat induced apoptosis in organoids, we assessed Caspase-3/7 activity. Molidustat-treated WT organoids showed no detectable activation, whereas approximately half of *Apc^fl/fl^* cells displayed increased cleaved Caspase-3/7 signal (Figure 6C–E), indicating apoptosis as a contributor to reduced viability consistent with activation of a synthetic lethal GSTP1-PHD2 vulnerability in APC-deficient epithelium. To determine whether the stem cell population was affected by Molidustat, we assayed the expression of *Lgr5* and found that mRNA expression was significantly reduced after treatment with the inhibitor, suggesting a reduction in the stem cell population (Figure 6F). To see whether this reduction of stem cells inhibited the ability to regenerate organoids, we conducted a clonogenicity assay from cells derived from vehicle or Molidustat-treated organoids. Consistent with the previous data, Molidustat significantly reduced the clonogenic potential of the organoid-derived cells, suggesting a reduction in the stem cell population (Figure 6G).

**Figure 6—figure supplement 1.**
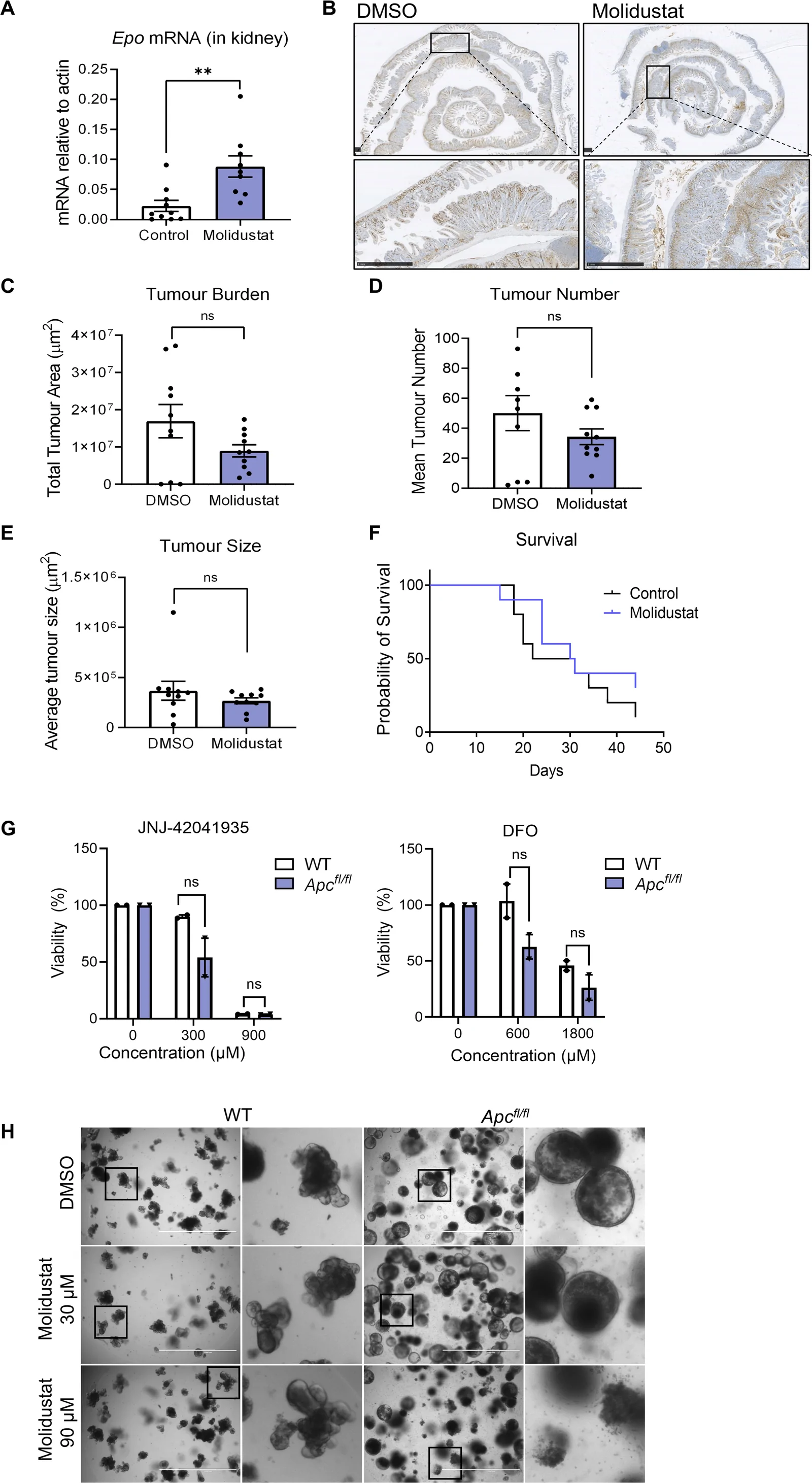
Evaluation of Molidustat in a colorectal cancer (CRC) in vivo model and effects of different prolyl hydroxylase domain (PHD) inhibitors on murine organoid viability. (**A**) *Epo* mRNA levels after Molidustat treatment. Mean + SEM is assessed using Mann-Whitney U test, **p<0.01. (**B**) Representative immunohistochemistry images of rolls of the upper section of the intestinal epithelium with a large number of macroscopic and microscopic tumours. Sections were stained with BrdU to label the DNA of actively dividing cells. Scale bar: 1000 μm. (**C**) Tumour burden of mice treated with DMSO or Molidustat. Mean + SEM is assessed using unpaired two-tailed Student’s t-test, (ns) non-significant. (**D**) Tumour number in mice treated with DMSO or Molidustat. Mean + SEM is assessed using unpaired two-tailed Student’s t-test, (ns) non-significant. (**E**) Tumour size of mice treated with DMSO or Molidustat. Mean + SEM is assessed using unpaired two-tailed Student’s t-test, (ns) non-significant. (**F**) Survival curve of mice treated with DMSO or Molidustat. Log-rank Mantel-Cox test, p=0.2924. N=10 DMSO, N=10 Molidustat. (**G**) Wild-type (WT) or *Apc^fl/fl^* organoid viability after 48 hr treatment with the indicated PHD inhibitors. Organoid viability percentage was assessed using an MTT assay. Mean + SEM is assessed using two-way ANOVA, (ns) non-significant. N=2 biologically independent experiments. (**H**) Representative images of WT and *Apc^fl/fl^* organoids treated with 30 and 90 μM Molidustat. Scale bar: 1000 μm. N=3 biologically independent experiments.

Overall, these findings demonstrate that Molidustat selectively reduces viability in organoids with distinct mutational backgrounds, particularly those with activated WNT signalling and/or KRAS pathway alterations, and that this effect is not reproduced by hydroxylase inhibition alone.

## Discussion

In this study, we identify GSTP1 as a previously unrecognised off-target interactor of Molidustat and reveal a synthetic lethal interaction between GSTP1 and PHD2 whose combined inhibition is selectively deleterious in APC-mutant CRC cells. Using TPP, chemical proteomics, CRISPR knockouts, and multi-omics profiling, we show that simultaneous inhibition of GSTP1 and PHD2 disrupts cellular homeostasis and induces oxidative stress and apoptosis, closely phenocopying the response to Molidustat treatment. Notably, GSTP1 and PHD2 loss each affect overlapping yet mechanistically distinct proteomic networks, providing a plausible basis for the emergent synthetic interaction. Synthetic lethal relationships of this type, where two enzymes regulate convergent pathways through independent mechanisms, are well documented, exemplified by BRCA1 and PARP in DNA-damage repair (Farmer et al., 2005; Dedes et al., 2011).

Mechanistically, our proteomic and metabolomic analyses indicate that the loss of dual GSTP1/PHD2 leads to ROS accumulation, elevated GSSG/GSH ratios, induction of oxidative-stress-associated apoptotic mediators, and downregulation of pro-survival pathways. Together, these coordinated alterations define a potential redox-collapse mechanism that underpins this synthetic interaction and explains the apoptotic phenotype observed upon both genetic double knockout and Molidustat exposure. However, the magnitude of oxidative stress suggests that additional oxidative-stress-associated cell death mechanisms may also contribute. While genetic rescue experiments would provide definitive confirmation of on-target specificity, the pronounced loss of fitness and apoptotic phenotype observed upon combined PHD2 and GSTP1 loss limited the feasibility of establishing stable rescued double knockout populations, thereby representing a limitation of the current study. Prior reports show that the pan-PHD inhibitor DMOG perturbs mitochondrial function before inducing HIF targets (Zhdanov et al., 2015), raising the possibility that GSTP1 may buffer mitochondrial stress in this context. Notably, hypoxia and HIF-targeting agents have recently been recognised as potential GSTP1 inhibitors based on molecular docking analyses, suggesting that GSTP1 engagement may represent a broader, previously underappreciated feature of compounds developed to modulate hypoxia signalling (Özcan and Ercan, 2025).

Although synthetic lethality is well established across cancer types, including examples such as PARP-BRCA, ATR-Wee1, and WRN in microsatellite-instable cancers (Farmer et al., 2005; Bukhari et al., 2019; Chan et al., 2019), the GSTP1-PHD2 interaction has not been previously described. This expands the spectrum of actionable dependencies in APC-mutant CRC. To fully validate this axis in vivo, two considerations are important. First, Molidustat delivery to the colon in our CRC models was suboptimal, but alternative strategies, such as implantable microdevices previously used to deliver PHD inhibitors directly into tumour xenografts (Price et al., 2019), provide feasible routes to achieve effective local exposure. Second, synthetic lethal relationships identified in vitro do not always translate to animal models (Zhang et al., 2025), underscoring the need for an APC-deficient CRC model with dual GSTP1/PHD2 loss. Together, these approaches will enable a rigorous assessment of the GSTP1-PHD2 dependency in vivo and strengthen the translational relevance of this vulnerability.

The clinical characteristics of Molidustat strengthen its translational potential. Long-term studies demonstrate good tolerability for up to 36 months in chronic kidney disease (Akizawa et al., 2019), an appealing safety profile given the toxicity of many frontline CRC treatments (Xie et al., 2020; Lee et al., 2014). Beyond CRC, Molidustat also exerts anti-tumour effects in breast cancer, where it reduces cell viability, induces cell-cycle arrest, enhances the activity of chemotherapeutic agents, and suppresses tumour growth in xenografts (Kachamakova-Trojanowska et al., 2020). Notably, Molidustat robustly stabilises HIF1α and induces VEGF to levels comparable to hypoxia, presenting an opportunity for combination strategies that counterbalance HIF-driven angiogenesis. Bevacizumab, which blocks VEGF-mediated angiogenic signalling (Manzi et al., 2023), represents one such rational partner. Molidustat also enhances the efficacy of gemcitabine, an antimetabolite that inhibits DNA synthesis, supporting exploration of this combination in additional cancer contexts (Kachamakova-Trojanowska et al., 2020; Pourquier et al., 2002). Furthermore, identification of PHD2 as a druggable dependency in KRAS-driven lung cancer (Reggiani et al., 2021) suggests potential synergy with KRAS inhibitors, including in APC/KRAS co-mutant CRC. By simultaneously exploiting the GSTP1-PHD2 synthetic lethal axis and modulating hypoxia signalling, Molidustat offers a mechanistically grounded entry point for multi-agent regimens that incorporate VEGF-pathway inhibitors, chemotherapeutics, or KRAS-targeted therapies.

A further consideration raised by our findings concerns the isoform selectivity of Molidustat. Although developed as a PHD2 inhibitor, Molidustat retains appreciable activity against PHD1 and PHD3 (Flamme et al., 2014), and the relative contributions of each isoform in the gut are non-redundant and, in some contexts, opposed. Genetic deletion of PHD1, but not PHD2 or PHD3, protects mice from DSS-induced colitis by limiting epithelial apoptosis and preserving barrier function, and PHD1 protein is elevated in mucosal biopsies from patients with ulcerative colitis and Crohn’s disease (Tambuwala et al., 2010). In colitis-associated cancer, PHD1 loss restrains tumour growth, whereas PHD2 haploinsufficiency has a complex phenotype resulting in increased proliferation, cell death, and modifies the immune response (Mamlouk et al., 2014). Notably, this pattern of isoform engagement is shared with other pan-PHD inhibitors that did not phenocopy Molidustat in our screens, indicating that PHD isoform profile alone is insufficient to explain its distinctive activity and pointing instead to the GSTP1 off-target engagement as the key distinguishing feature. Nonetheless, the compartment-specific roles of PHD1 and PHD2 argue for localised colonic delivery strategies, such as the microdevice-based approaches discussed above, which would concentrate Molidustat at the APC-mutant epithelium to exploit the GSTP1-PHD2 synthetic lethal axis while limiting systemic exposure and the potentially deleterious consequences of systemic PHD2 inhibition.

Several GSTP1 inhibitors are currently in pre-clinical development, and our findings suggest that their antineoplastic activity may be potentiated through concurrent disruption of PHD2. Thus, a dual-inhibition strategy targeting both GSTP1 and PHD2 may yield synergistic efficacy, particularly in genetic backgrounds that rely on coordinated redox and hypoxia adaptation programmes. In this context, Molidustat, despite its originally intended selectivity towards PHD2, emerges as a versatile candidate for rational combination approaches, not only due to its clinical tractability but also because of its unexpected engagement of GSTP1. Collectively, these observations broaden the therapeutic landscape for exploiting oxidative-stress vulnerabilities across multiple cancer subtypes.

## Data Availability

The MS proteomics data generated in this study have been deposited to the ProteomeXchange Consortium via the PRIDE partner repository under accession number PXD071906. The metabolomics data have been deposited to the MassIVE repository under accession number MSV000103644. Original, uncropped images for all western blots shown in the figures and figure supplements are provided as Source Data files. All other data supporting the conclusions are available in the article, its figure supplements, and its supplementary files. The following datasets were generated: AgataM Alex vonK 2026Molidustat Targets a Synthetic Lethal Vulnerability in APC-Mutant Colorectal Cancer through GSTP1 and PHD2 Co-InhibitionPRIDEPXD07190610.7554/eLife.110991PMC1375079643007703 Marques JuniorJ von KriegsheimA 2026MassIVE MSV000103644 FilesMassIVEMSV000103644

## Additional files

Supplementary file 1. Hits from the thermal proteome profiling (TPP) screen (Easy50 method). (XLSX).

Supplementary file 2. IOX4 screen mass spectrometry proteomics: FragPipe search output and analysis files. (XLSX).

Supplementary file 3. HT29 mass spectrometry proteomics: DIA-NN search output and analysis files. (XLSX).

Supplementary file 4. HT29 mass spectrometry metabolomics: search output and analysis files. (XLSX).

MDAR checklist
