# Supplementary material for: Molidustat targets a synthetic lethal vulnerability in APC-mutant colorectal cancer through GSTP1 and PHD2 co-inhibition
Source: eLife. 2026 Nov 10;15:RP110991. doi: 10.7554/eLife.110991 (PMC13750796; doi:10.7554/eLife.110991)
Supplement: Supplementary file 1 [file elife-110991-fig1-data1.zip › Figure 1-source data 1/Figure 1-source data 1.pdf]

Figure 1C: PHD2

Annotated Source | Molecular Weight Marker

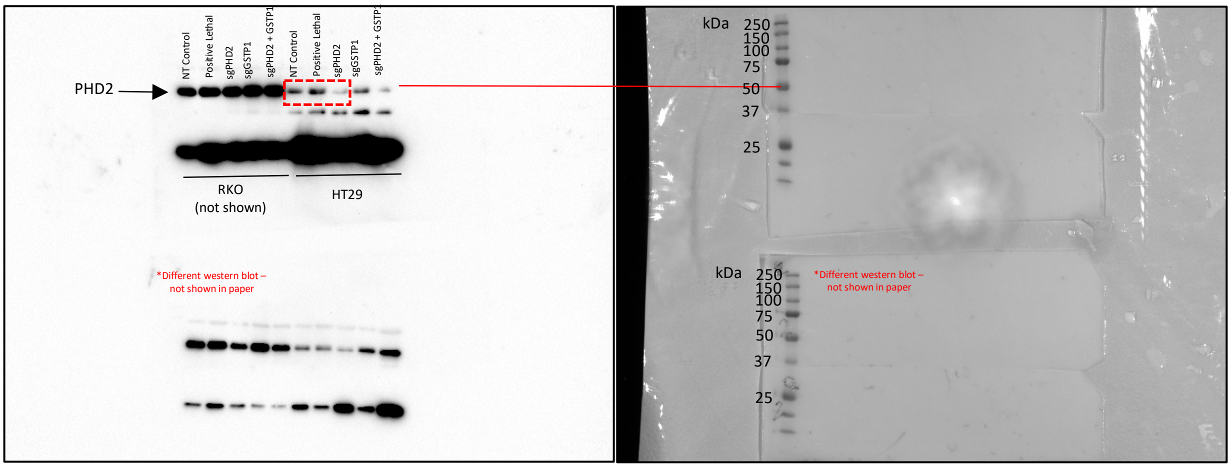

Bands shown in paper

Figure 1C: Actin

Annotated Source | Molecular Weight Marker

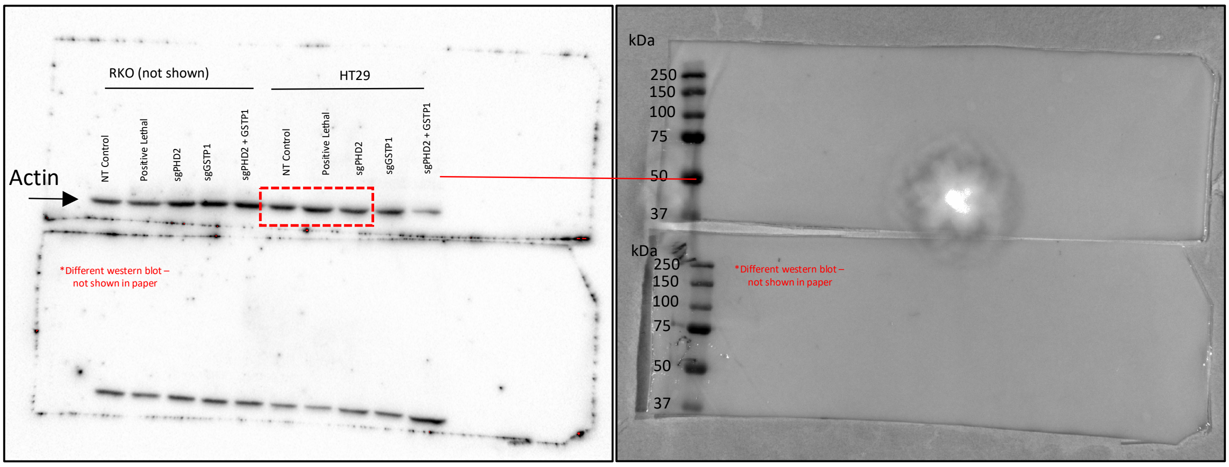

Bands shown in paper
