# Supplementary material for: Molidustat targets a synthetic lethal vulnerability in APC-mutant colorectal cancer through GSTP1 and PHD2 co-inhibition
Source: eLife. 2026 Nov 10;15:RP110991. doi: 10.7554/eLife.110991 (PMC13750796; doi:10.7554/eLife.110991)
Supplement: Supplementary file 3 [file elife-110991-fig1-figsupp1-data1.zip › Figure 1-figure supplement 1-source data 1/Figure 1-figure supplement 1-source data 1.pdf]

Figure 1—figure supplement 1A and B: HIF1α

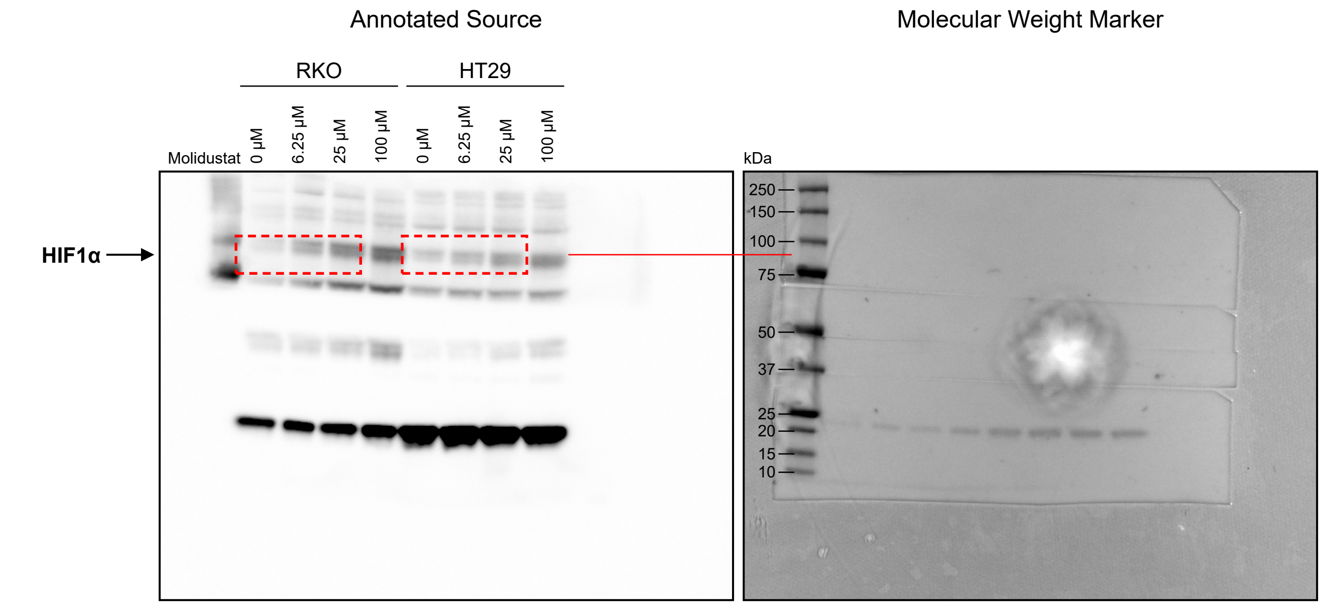

Figure 1—figure supplement 1A and B: PHD2

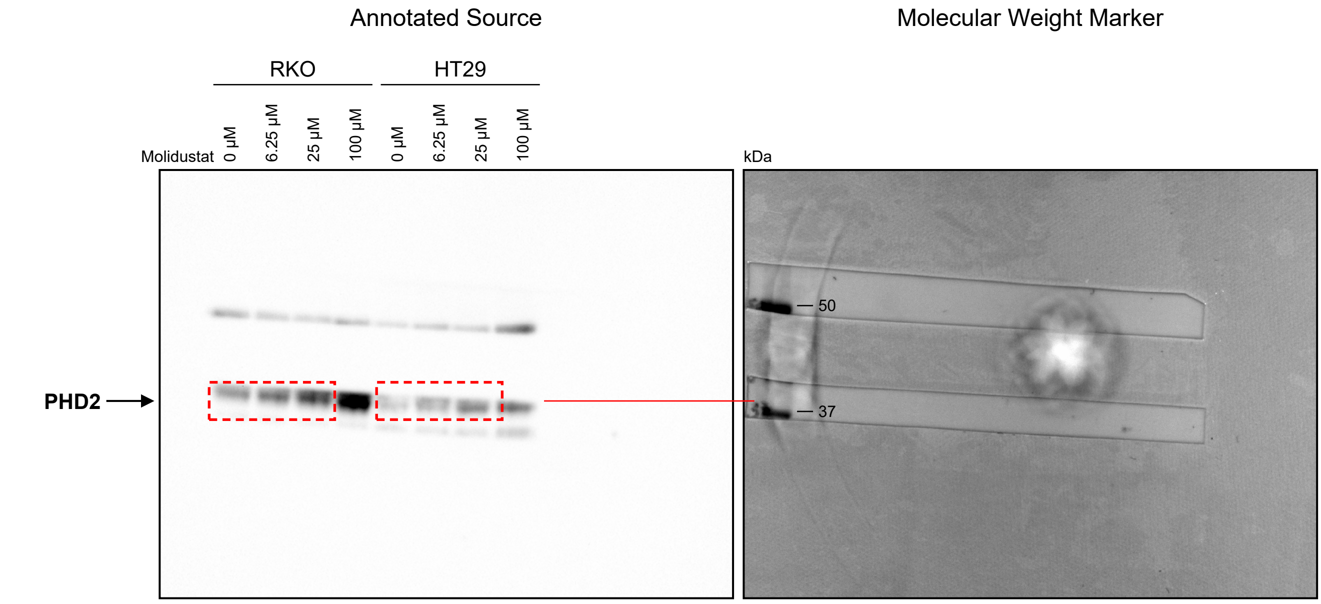

PHD2 and β-tubulin were probed on horizontal strips cut from the same membrane; the PHD2 and β-tubulin images are exposures of that membrane, and the MW marker image is shared.

Figure 1—figure supplement 1A and B: β-Tubulin

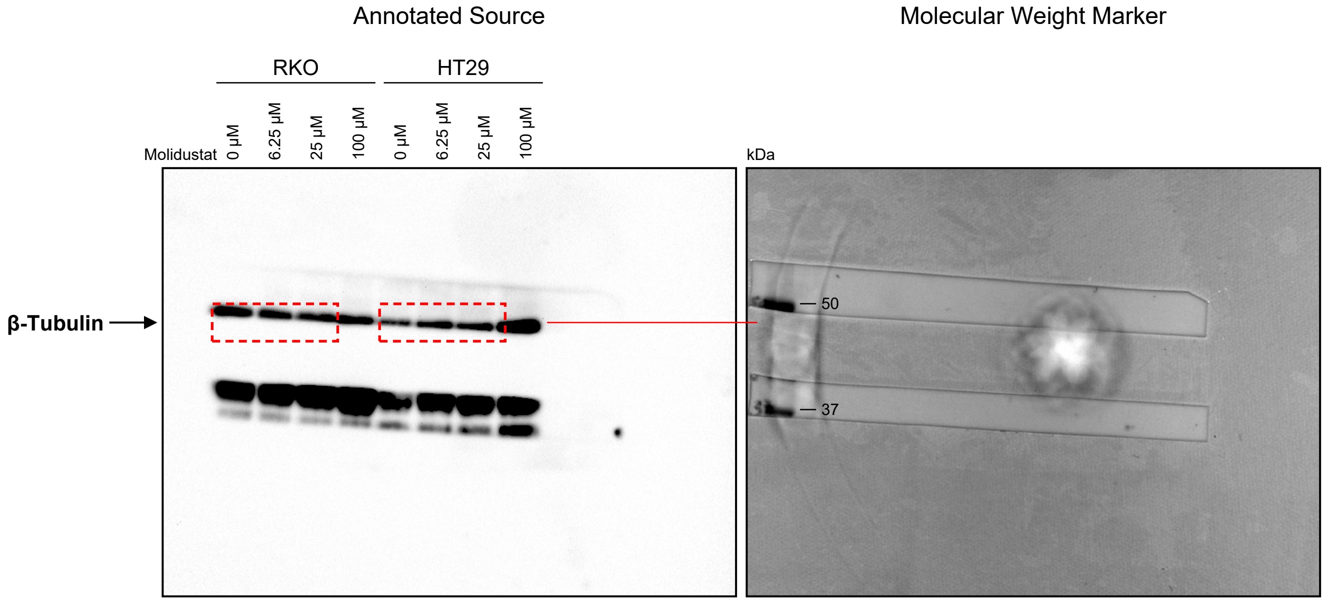

Bands shown in paper
