# Supplementary material for: Molidustat targets a synthetic lethal vulnerability in APC-mutant colorectal cancer through GSTP1 and PHD2 co-inhibition
Source: eLife. 2026 Nov 10;15:RP110991. doi: 10.7554/eLife.110991 (PMC13750796; doi:10.7554/eLife.110991)
Supplement: Supplementary file 5 [file elife-110991-fig3-data1.zip › Figure 3-source data 1/Figure 3-source data 1.pdf]

Figure 3B:

Annotated Source

Molecular Weight Marker

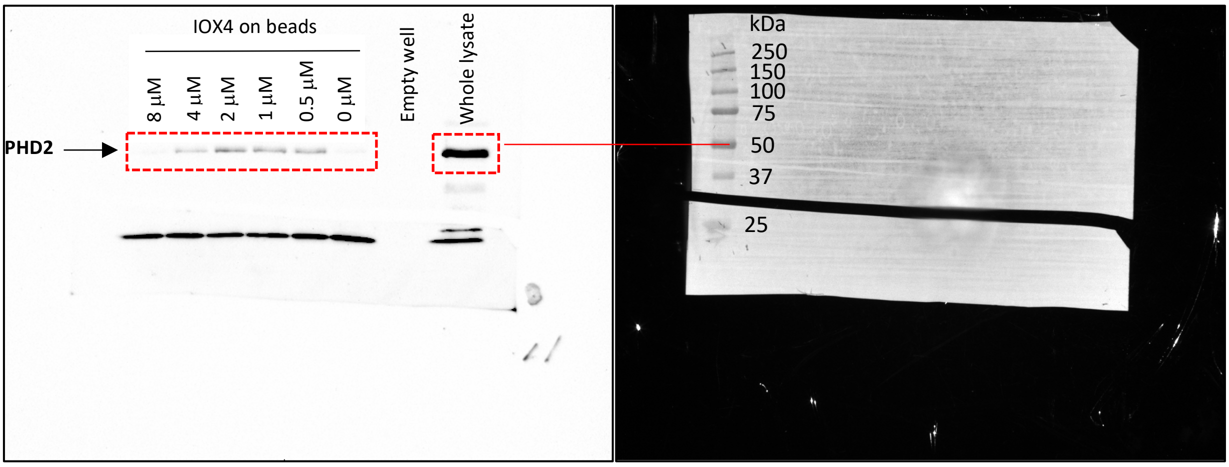

Bands shown in paper

Figure 3C:

Annotated Source

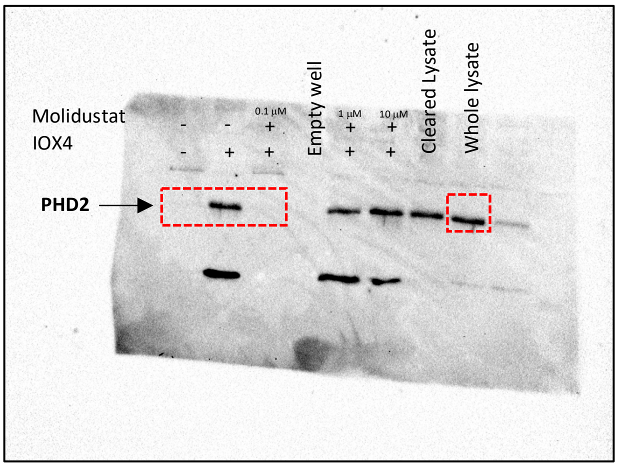

Bands shown in paper

Figure 3D:

Annotated Source

Molecular Weight Marker

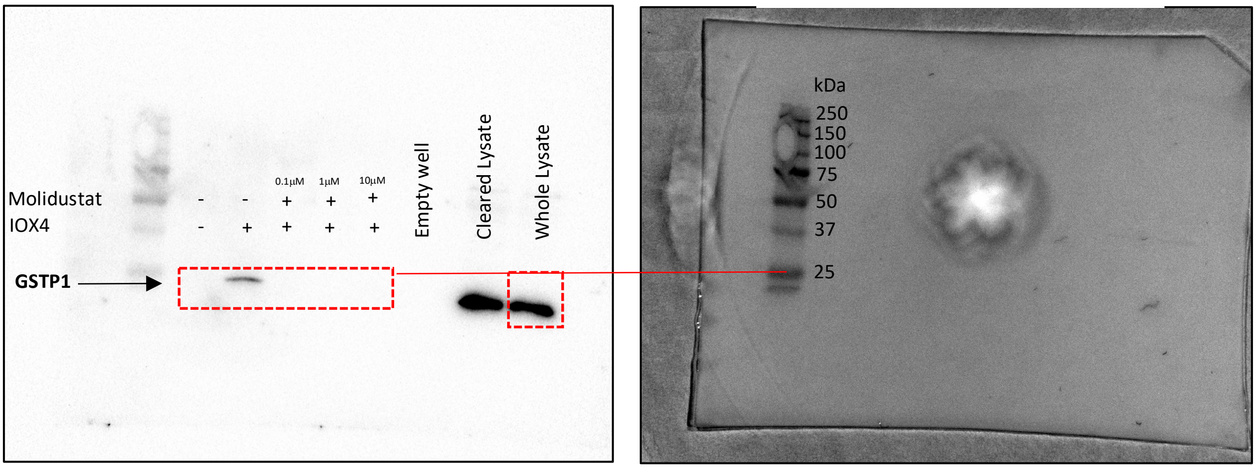

Bands shown in paper
