# Supplementary material for: Molidustat targets a synthetic lethal vulnerability in APC-mutant colorectal cancer through GSTP1 and PHD2 co-inhibition
Source: eLife. 2026 Nov 10;15:RP110991. doi: 10.7554/eLife.110991 (PMC13750796; doi:10.7554/eLife.110991)
Supplement: Peer review history

# Editor report: eLife Assessment

**DOI:** 10.7554/eLife.110991.3.sa0

**Authors:** Qing Zhang

**Affiliations:** University of Texas Southwestern Medical Center United States

**Keywords:** Compelling, Important

This **important** paper substantially advances our understanding of how Molidustat may work, beyond its canonical role, by identifying its therapeutic targets in cancer. This study presents a **compelling** and well-structured investigation into the therapeutic vulnerabilities of APC-mutant colorectal cancer. This work will be of broad interest to the cancer community in studying small molecules and their therapeutic targets.

# Referee report: Reviewer #1 (Public review)

**DOI:** 10.7554/eLife.110991.3.sa1

[Editor's note: this version has been assessed by the Reviewing Editor without further input from the original reviewers. The authors have addressed the comments raised in the previous round of review.]

Summary:

The authors aimed to uncover novel therapeutic vulnerabilities in APC-mutant colorectal cancer (CRC), which constitutes the majority of CRC cases. They hypothesized that modulating oxygen-sensing pathways (via PHD inhibition) could disrupt adaptive stress responses in these tumours.

Strengths:

The study employs a powerful, two-pronged approach to identify Molidustat's targets. By using both Thermal Proteome Profiling (TPP) and an orthogonal chemical proteomic competition assay, the authors provide compelling evidence that GSTP1 is a genuine, direct off-target, effectively addressing the common limitation of indirect effects in proteomic screens.

# Referee report: Reviewer #2 (Public review)

**DOI:** 10.7554/eLife.110991.3.sa2

Summary:

The authors aimed to determine Molidustat targets and the potential utility of these findings. They clearly demonstrate that Molidustat interferes with GSTP1 and some other proteins on top of PHD2. They also demonstrate that PHD2 deletion is not sufficient to recapitulate Molidustat effects in cells and proteomes. Finally, they demonstrate synthetic lethality in organoids for Molidustat and APC deletion.

Strengths:

The data on Molidustat proteomes, GSTP1 binding, inhibition and metabolic health of organoids is really clear. All biochemical, docking and omic data are really strong. The potential impact of these findings could be the use of Molidustat in APC null tumours and awareness of potential off-target effects.

# Referee report: Reviewer #3 (Public review)

**DOI:** 10.7554/eLife.110991.3.sa3

In this paper, the authors revealed that Molidustat can induce a dose-dependent increase in Caspase-3/7 activity in the HT29 cell line, which is an APC-mutant colorectal cancer cell line. More importantly, they found that targeting PHD2 alone cannot cause cell death. By using thermal proteome profiling (TPP) and orthogonal chemical proteomic competition assays, they determined GTSP1 as a previously undiscovered off-target of Molidustat. They also revealed that combined PHD2 and GSTP1 loss leads to an increase in intracellular ROS and apoptosis. Moreover, they evaluated the effects of Molidustat in colonic organoids and showed that Molidustat has a high selectivity for colonic organoids with activated WNT signaling and/or KRAS pathway alterations, and this effect is not reproduced by hydroxylase inhibition alone, providing a new potential approach to targeting both PHD2 and GTSP1 for the treatment of APC-mutant CRC.

# Author comment: Author response

**DOI:** 10.7554/eLife.110991.3.sa4

**Authors:** Chiara Asselborn, Agata N Makar, Jair G Marques Junior, Aslihan Bastem Akan, Athanasia Yiapanas, Carrie Jennings, Ana Perez-Lopez, Jimi Wills, Asier Unciti-Broceta, Kevin B Myant, Alex von Kriegsheim

**Affiliations:** University of Edinburgh Edinburgh United Kingdom; University of Edinburgh Edinburgh United Kingdom; University of Edinburgh Edinburgh United Kingdom; University of Edinburgh Edinburgh United Kingdom; University of Edinburgh Edinburgh United Kingdom; University of Edinburgh Edinburgh United Kingdom; Technische Universität Berlin Berlin Germany; University of Edinburgh Edinburgh United Kingdom; University of Edinburgh Edinburgh United Kingdom; University of Edinburgh Edinburgh United Kingdom; University of Edinburgh Edinburgh United Kingdom

The following is the authors’ response to the original reviews.

**Public Reviews:**

**Reviewer #1 (Public review):**

Summary:

The authors aimed to uncover novel therapeutic vulnerabilities in APC-mutant colorectal cancer (CRC), which constitutes the majority of CRC cases. They hypothesized that modulating oxygen-sensing pathways (via PHD inhibition) could disrupt adaptive stress responses in these tumours.

Strengths:

The study employs a powerful, two-pronged approach to identify Molidustat's targets. By using both Thermal Proteome Profiling (TPP) and an orthogonal chemical proteomic competition assay, the authors provide compelling evidence that GSTP1 is a genuine, direct off-target, effectively addressing the common limitation of indirect effects in proteomic screens.

Weaknesses:

(1) In Figure 1, the current data rely on a single guide RNA (sgRNA). To make the data solid, at least two independent sgRNAs targeting different regions of PHD2 should be used.

We thank the reviewer for raising this. Clarity on the CRISPR strategy was missing from the original submission and we have now added the following to the Methods (Page 4). We did not use a single sgRNA. PHD2 was targeted with a pool of three chemically modified crRNAs:

(IDT Alt-R; target sequences: 5'-TACAACCAGCATATGCTACA, 5'GTGGCTGCCGAAGCCGAGCC, 5'-GATAAGATCACCTGGATCGA)

Delivered as in vitro assembled ribonucleoprotein complexes with high-fidelity Cas9. This format has been reported to achieve high on-target efficiency while minimising off-target cutting [1,2] such that any residual stochastic off-target events are distributed across the population and are not expected to manifest as a coherent phenotype at the population level. Working with pooled, unselected knockouts rather than single-cell clones also avoids the confounds of clonal heterogeneity that normally motivate the use of multiple independent guides and rescue experiments in single-clone workflows. We have previously validated this approach for GSTP1 knockout in a separate single-cell proteomics study [3], where loss of GSTP1 protein was observed in over 90% of single cells and GSTP1 was the most significantly altered protein between sgControl and sgGSTP1 populations.

(2) Figure 3E: Asn205 site should be mutated to prove that whether Molidustat inhibits GSTP1 activity via Asn205 or not.

This is a good suggestion, and we explored it *in silico* before concluding it was not tractable. We used PyMol mutagenesis to model Molidustat binding to GSTP1 variants at the predicted contact residues: Asn205 was mutated to Ala, Gly and Ser; Trp39 (predicted to hydrogen-bond Molidustat) was mutated to Ala, Phe and Thr; and a Tyr8Phe/Asn205Ser double mutant was also modelled. In every case, Molidustat reoriented within the active site and adopted an alternative hydrogen-bonding configuration (most commonly with Tyr8), yielding a docking score equal to or better than binding to native GSTP1 (Author response image 1– Author response image 4). The model therefore does not predict any single or double point mutant that would ablate Molidustat binding in a clean, interpretable way, and we could not design a rational loss-of-interaction mutant on this basis. Given this limitation, and that definitive mapping of the binding interface would require co-crystallography, which is beyond the scope of the present study, we have moved the docking model to the supplement and flagged it as predictive rather than definitive.

**Author response image 1.** Molidustat in native GSTP1.

**Author response image 2.** Molidustat docking with mutated GSTP1, Asn205 mutated to Gln205.

**Author response image 3.** Molidustat docking with mutated GSTP1, Tyr39 mutated to Phe39.

**Author response image 4.** Molidustat docking with mutated GSTP1, Asn205 mutated to Ser205 and Tyr8 mutated to Phe8.

(3) Figure 5B and 5C: The metabolic imbalance phenotype observed upon dual knockout of PHD2 and GSTP1 requires rescue experiments to confirm on-target specificity.

We thank the reviewer for this important point and agree that rescue experiments could represent the most direct demonstration of on-target specificity for the metabolic phenotype observed in Figures 5B and 5C. These rescue experiments are necessary when working with single clones, as they allow for comparing a knock-out clone with a reconstituted pool and sidestep the issue of clonal heterogeneity.

In our case, we think that there is no advantage to doing so, as we work with pooled knockouts, so any clonal heterogeneity is diluted in the pool.

One could even make the case that such a rescue experiment would introduce additional artefacts. Combined loss of PHD2 and GSTP1 leads to reduced cellular viability, with decreased proliferation and increased apoptosis, consistent with a synthetic lethal interaction. To devise a rescue experiment, we would have to isolate a single-cell clone (the pool is not a complete 100% knock out, WT cells would outgrow the knock out cells). The isolation of such a clone that has overcome the anti-proliferative insult of the double knockout is likely to have a phenotype distinct from the original, pooled population, as would the rescued have from the WT cells. For these reasons, we have not performed rescue experiments in the current study. We have added the absence of a rescue as a limitation to the study in the discussion

“While genetic rescue experiments would provide definitive confirmation of on-target specificity, the pronounced loss-of-fitness and apoptotic phenotype observed upon combined PHD2 and GSTP1 loss limited the feasibility of establishing stable rescued double-knockout populations, and therefore represents a limitation of the current study.”

**Reviewer #2 (Public review):**

Summary:

The authors aimed to determine Molidustat targets and the potential utility of these findings. They clearly demonstrate that Molidustat interferes with GSTP1 and some other proteins on top of PHD2. They also demonstrate that PHD2 deletion is not sufficient to recapitulate Molidustat effects in cells and proteomes. Finally, they demonstrate synthetic lethality in organoids for Molidustat and APC deletion.

Strengths:

The data on Molidustat proteomes, GSTP1 binding, inhibition and metabolic health of organoids is really clear. All biochemical, docking and omic data are really strong. The potential impact of these findings could be the use of Molidustat in APC null tumours and awareness of potential off-target effects.

Weaknesses:

A main but minor weakness is that Molidustat also inhibits other PHDs, although these are less expressed. PHD1 has been shown to control the cell cycle and be expressed in the colon, where it is needed for viability. Although this does not explain the lack of effect of other PHD inhibitors, it does warrant some discussion. The use of MTT is not very good to detect viability when it measures metabolism; this also needs to be discussed and perhaps supplemented with colony or cell number measurements.

Great point, for this reason, we have assayed apoptosis throughout. In addition, we have added a clonogenicity assay with APC organoids. Organoid cells were treated with an acute dose of Molidustat. We subsequently measured the level of Lgr5 (a stem cell marker) and of the ability of the cells to generate organoids (these data have been added as Figure 5 F-G.)

**Reviewer #3 (Public review):**

In this paper, the authors revealed that Molidustat can induce a dose-dependent increase in Caspase-3/7 activity in the HT29 cell line, which is an APC-mutant colorectal cancer cell line. More importantly, they found that targeting PHD2 alone cannot cause cell death. By using thermal proteome profiling (TPP) and orthogonal chemical proteomic competition assays, they determined GTSP1 as a previously undiscovered off-target of Molidustat. They also revealed that combined PHD2 and GSTP1 loss leads to an increase in intracellular ROS and apoptosis. Moreover, they evaluated the effects of Molidustat in colonic organoids and showed that

Molidustat has a high selectivity for colonic organoids with activated WNT signaling and/or KRAS pathway alterations, and this effect is not reproduced by hydroxylase inhibition alone, providing a new potential approach to targeting both PHD2 and GTSP1 for the treatment of APC-mutant CRC.

Specific comments:

(1) What is the possible molecular mechanism of dual GSTP1/PHD2 loss, inducing cell death?

This is an important question. Our data support a model in which combined loss of GSTP1 and PHD2 disrupts cellular redox homeostasis, leading to accumulation of reactive oxygen species, increased GSSG/GSH ratios, and depletion of antioxidant buffering capacity. This redox imbalance is accompanied by downregulation of pro-survival pathways. In this context, activation of apoptotic signalling, as evidenced by increased caspase-3/7 activity and proteomic enrichment of apoptosis-associated pathways, contributes to the observed cell death phenotype.

While apoptosis is supported by our data, the magnitude of oxidative stress suggests that additional oxidative stress-associated cell death mechanisms may also contribute. We have clarified this point in the Discussion (Page 11).

(2) Can the authors mutate the binding site of Molidustat on GTSP1 to verify the in silico docking results?

This is a very important question. Currently, the model is of limited value. Reviewer 1 had a similar question. Can we refer you to Reviewer 1, question 2.

(3) Evidence for Molidustat inhibiting PHD2 activity or stabilising HIF-1α should be provided.

We thank the reviewer for this suggestion. Data showing HIF-1α stabilisation and evidence of downstream signalling is now added to Supplementary Figure 1.

**Recommendations for the authors:**

**Reviewer #2 (Recommendations for the authors):**

I only have minor suggestions:

Molidustat also inhibits other PHDs, although these are less expressed. PHD1 has been shown to control the cell cycle and be expressed in the colon, where it is needed for viability. Although this does not explain the lack of effect of other PHD inhibitors, it does warrant some discussion. The use of MTT is not very good to detect viability when it measures metabolism; this also needs to be discussed and perhaps supplemented with colony or cell number measurements.

This is correct, PHD1 is of particular interest, given the effects inhibition/knock-out has on the inflamed colon. We have added a new paragraph to the Discussion (Page 13) that addresses the isoform selectivity of Molidustat. We note that, although developed as a PHD2 inhibitor, Molidustat retains appreciable activity against PHD1 and PHD3 [4], and we discuss the non-redundant and in some contexts opposing roles of PHD1 and PHD2 in the colon, PHD1 loss is protective in DSS colitis [5] and restrains colitis-associated tumour growth, whereas PHD2 loss in the tumour and stroma is reported to inhibit metastasis and treatment response [6]. We further note that this pattern of isoform engagement is shared with other pan-PHD inhibitors that did not phenocopy Molidustat in our screens, indicating that PHD isoform profile alone is insufficient to explain Molidustat’s distinctive activity and pointing to GSTP1 off-target engagement as the key distinguishing feature. We argue that localised colonic delivery (as discussed earlier in the Discussion) would concentrate drug at the APC-mutant epithelium while limiting systemic exposure.

We fully agree with the reviewer, MTT measures metabolic activity/NADH levels rather than viability in the strict sense, and that this is particularly relevant for a compound that perturbs redox metabolism. We have added a clonogenicity assay in APC organoids (Fig. 5 F-G) to supplement the MTT and Cleaved Caspase 3 assays already present in the manuscript.

(1) Lee, J. K. et al. Directed evolution of CRISPR-Cas9 to increase its specificity. Nat. Commun. 9, (2018).

(2) Sakovina, L., Vokhtantsev, I., Vorobyeva, M., Vorobyev, P. & Novopashina, D. Improving Stability and Specificity of CRISPR/Cas9 System by Selective Modification of Guide RNAs with 2′-fluoro and Locked Nucleic Acid Nucleotides. Int. J. Mol. Sci. 23, (2022).

(3) Makar, A. N., Holkham, J., Lilla, S., Wilkinson, S. & von Kriegsheim, A. Overcoming preservation challenges to enable single-cell proteomics of fixed cell and tissue samples with retained proteome integrity. Preprint at https://doi.org/10.1101/2025.03.10.642380 (2025).

(4) Flamme, I. et al. Mimicking hypoxia to treat anemia: HIF-stabilizer BAY 85-3934 (molidustat) stimulates erythropoietin production without hypertensive effects. PLoS One 9, (2014).

(5) Tambuwala, M. M. et al. Loss of prolyl hydroxylase-1 protects against colitis through reduced epithelial cell apoptosis and increased barrier function. Gastroenterology 139, (2010).

(6) Leite de Oliveira, R. et al. Gene-Targeting of Phd2 Improves Tumor Response to Chemotherapy and Prevents Side-Toxicity. Cancer Cell 22, (2012).
